# A systematic review and meta-analysis of the association between low socioeconomic status and all-cause mortality in patients with diabetes

**DOI:** 10.3389/fpubh.2026.1841893

**Published:** 2026-06-26

**Authors:** Joanna Konkol, Chunrong Pang, Cheng Pu

**Affiliations:** 1 Chengdu University of Traditional Chinese Medicine, Chengdu, China; 2 Department of Infectious Diseases and Public Health, City University of Hong Kong, Hong Kong, Hong Kong SAR, China; 3 Chengdu University of Traditional Chinese Medicine School of Basic Medicine, Chengdu, China

**Keywords:** all-cause mortality, diabetes mellitus, health inequalities, meta-analysis, socioeconomic status

## Abstract

**Objective:**

The aim of this study was to evaluate the association between socioeconomic status (SES) and all-cause mortality among individuals with diabetes. We also examined how individual SES components, including income, employment, education, and housing conditions, were associated with mortality risk.

**Methods:**

Following PRISMA 2020 guidelines, we searched PubMed, Embase, the Cochrane Library, and Web of Science through September 2025. Eligible studies included adults with type 1 or type 2 diabetes reporting associations between SES indicators (income, education, occupation, or area-level deprivation) and all-cause mortality. Pooled odds ratios (ORs) with 95% confidence intervals (CIs) were calculated using fixed- or random-effects models based on heterogeneity (I^2^). Publication bias was assessed using funnel plots and Egger’s test.

**Results:**

Nineteen studies were included. Low SES was associated with a higher risk of all-cause mortality (OR = 1.67; 95% CI: 1.49–1.88; *p* < 0.00001). Similar associations were observed across income, education, housing, and employment domains. Heterogeneity was substantial (*I*^2^ = 99%), indicating considerable variability across studies. Sensitivity analyses showed that no single study materially influenced the pooled estimate, and publication bias appeared minimal.

**Conclusion:**

Low socioeconomic status is associated with increased mortality among individuals with diabetes. Addressing socioeconomic inequalities through improved access to education, employment opportunities, healthy environments, and equitable healthcare may help reduce survival disparities and mitigate the overall burden of diabetes.

**Systematic review registration:**

https://www.crd.york.ac.uk/prospero/display_record.php?RecordID=1248984, identifier PROSPERO (CRD420251248984).

## 1 Introduction

An estimated 589 million adults worldwide are living with diabetes mellitus (DM), with projections indicating an increase to 853 million by 2050 (1), making it a major public health concern. Despite improvements in diagnosis and treatment, this disease continues to contribute to premature all-cause mortality. Large population-based studies show that individuals with diabetes have a significantly higher risk of all-cause mortality than those without diabetes (2, 3). This is mainly due to the increased risk of cardiovascular disease, kidney disease, respiratory disorders, and infections (4). Similar observations have been reported in different populations worldwide (5). The increase in diabetes prevalence has been particularly rapid in low- and middle-income countries, where the increase in incidence has outpaced that observed in high-income settings (6). In line with this, Global Burden of Disease analyses show that mortality attributable to diabetes is disproportionately higher in regions with lower levels of economic development (7), highlighting the potential contribution of socioeconomic disadvantage to inequalities in both diabetes burden and survival outcomes. Additionally, type 2 diabetes is associated with a high burden of comorbid conditions, with cardiovascular, musculoskeletal, and mental health disorders among the most common, and population-based studies indicate that multimorbidity is highly prevalent in people with diabetes, which may considerably complicate disease management (8). The social determinants of health (SDOH), which the World Health Organization defines as “the conditions in which people are born, grow, live, work, and age, and the wider set of forces and systems shaping these conditions,” have a significant impact on health disparities. These determinants contribute to preventable health inequalities and are estimated to account for approximately 30–55% of health outcomes (9). Socioeconomic status (SES), typically measured by education, income, and occupation, represents one of the key determinants (10).

Lower SES has been linked to a higher prevalence of diabetes, limited access to preventive care, and poorer disease management outcomes (10–12). Individuals from disadvantaged backgrounds more often face financial hardship, inadequate housing, limited healthcare access, and lower levels of education, all of which have been linked to difficulties in both prevention and treatment (10). Large-scale analyses further indicate that lower education, income, and occupational status are associated with a 30–40% higher risk of developing type 2 diabetes, a pattern observed across multiple national datasets, including NHANES (12, 13). In addition, longitudinal studies have shown that income inadequacy during childhood is associated with an increased risk of diabetes in adulthood (14).

Once diabetes develops, socioeconomic differences continue to influence disease management and, consequently, patient survival. Previous studies have shown that lower SES is associated with higher all-cause mortality among individuals with diabetes, even after adjustment for lifestyle and clinical factors (15). For instance, in the UK, patients from the most deprived quintile had a 40–50% higher risk of premature death compared with those from the least deprived areas (15), with similar patterns reported in Scotland, particularly among men (16). Among the main SES components—income, education, employment, and housing—each is linked to higher mortality in individuals with diabetes. In Korea, very low income more than doubled the risk of mortality, particularly among younger men (17). A similar but less pronounced pattern was observed for education, with lower educational attainment being associated with approximately 1.5–1.7 times higher mortality (18). Employment instability is also associated with worse outcomes. Job loss or unemployment increased mortality risk by up to tenfold among women with diabetes (19). In addition, studies on housing deprivation have shown that mortality was highest among tenants and individuals living in low-quality housing, even after adjustment for income and education (20). Given existing evidence suggesting an association between SES and mortality in individuals with diabetes, we aimed to quantitatively synthesize available studies to quantify the strength of the association between low SES and all-cause mortality. In addition, we explored the relative contribution of different SES components and examined potential moderators, including sex, SES domain, study design, and geographic context. In this context, our findings may provide evidence to support policies aimed at reducing socioeconomic inequalities in diabetes outcomes.

## 2 Methods

### 2.1 Literature search

This study was registered in PROSPERO (CRD420251248984) and complied with PRISMA 2020 guidelines (21) (see Supplementary Table S1). Four databases (PubMed, Cochrane, Embase, and Web of Science) were used for the literature search. The keywords “Diabetes Mellitus,” “Socioeconomic Factors,” and “Mortality” were used to find eligible records. Studies published up until September 11, 2025, were included in the search. We provided the full search strategy in Supplementary Table S1. Two independent reviewers screened all titles and abstracts to identify eligible studies. Discrepancies were resolved through discussion and consensus.

### 2.2 Inclusion and exclusion criteria

Eligibility criteria were defined according to the PECOS framework:

- Population (P): adults (≥18 years) with type 1 or type 2 diabetes mellitus.
- Exposure (E): low socioeconomic status (income, education, or occupation).
- Comparator (C): higher socioeconomic status groups.
- Outcome (O): all-cause mortality (hazard ratio, risk ratio or odds ratio).
- Study design (S): prospective or retrospective cohort studies, case–control studies, or randomized controlled trials reporting SES–mortality associations.

Inclusion criteria:

1. Original observational studies (prospective or retrospective cohort, case–control)
2. Reported the association between low SES and mortality (providing HR/RR/OR with 95% CI)
3. Study population consisted of patients with diabetes

Exclusion criteria:

1. Reviews, commentaries, abstracts, or other non-original studies
2. Studies without mortality outcomes or without SES exposure data
3. Studies with incomplete data or of low methodological quality

### 2.3 Data extraction

Two independent researchers (JK and CP) conducted the data extraction. Any disagreements were resolved through discussion to ensure consistency. Extracted data included the following:

1. Study characteristics: first author, year of publication, study country, and study design.
2. Population details: sample size, gender, and age.
3. Outcomes and effect measures: adjustment variables, outcomes, and effect sizes (OR, RR, HR) with 95% confidence intervals (CIs).

### 2.4 Quality assessment

The methodological quality and risk of bias of each included study were evaluated using standardized tools according to study design. For randomized controlled trials (RCTs), we applied the Revised Cochrane Risk-of-Bias Tool (RoB 2), which assesses bias across five domains: the randomization process, deviations from intended interventions, missing outcome data, measurement of the outcome, selection of the reported result.

For non-randomized studies, including self-controlled studies and retrospective studies, the Risk of Bias in Non-Randomized Studies of Interventions (ROBINS-I) tool was used. Seven domains of bias were assessed, including: confounding, selection of participants, classification of interventions, deviations from intended interventions, missing data, measurement of outcomes, and selection of reported results.

The robvis web application^1^ was used to visualize risk-of-bias assessments. It produced summary bar charts and traffic-light plots to show which studies classified as having a low, unclear, or high risk of bias within each domain.

### 2.5 Statistical analysis

All of the analyses were performed using Review Manager version 5.4.1 (Cochrane Collaboration, Oxford, United Kingdom). Odds ratios (OR) with 95% confidence intervals (CIs) were used to represent effect sizes. The I^2^ statistic was used to assess statistical heterogeneity between studies; significant heterogeneity was defined as *p* < 0.05 or I^2^ > 50%. When there was substantial heterogeneity, a random-effects model was utilized; otherwise, a fixed-effect model was employed. Funnel plots were used to visually assess publication bias when at least 10 studies were included. In addition, Egger’s regression test was conducted in Stata version 15.0 (StataCorp, College Station, TX, United States) to evaluate asymmetry. All statistical tests were two-sided, and *p* < 0.05 was considered statistically significant.

## 3 Results

### 3.1 Study characteristics and results of the screening process

**Figure 1:**
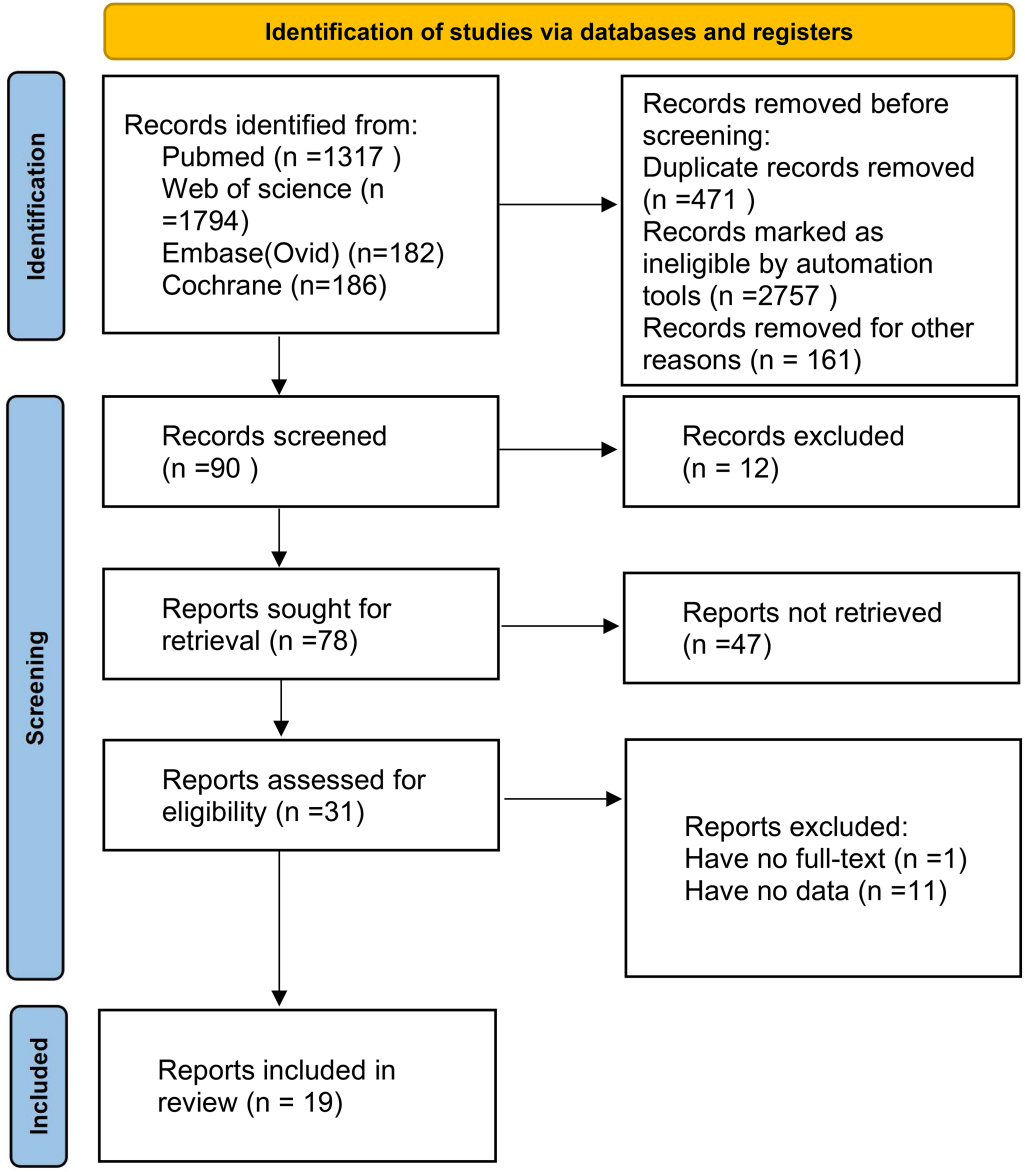
PRISMA flow diagram of study selection.

A total of 3,479 records were identified: 1,317 from PubMed, 1,794 from Web of Science, 182 from Embase (Ovid), and 186 from the Cochrane Library. After removing 471 duplicates, 2,989 records were excluded based on ineligible article types, irrelevant topic, lack of full text, or missing data. Finally, 19 studies met the inclusion criteria and were included in the meta-analysis (Figure 1) (17, 19, 20, 22–37). The included studies were conducted across multiple regions, including Europe, North America, Asia, and Australia, with the largest number originating from Korea, the United Kingdom, China, and the United States. Most studies were observational cohort studies, whereas only one randomized controlled trial was included. The analyzed populations included both type 1 and type 2 diabetes, although type 2 diabetes predominated across studies. The key characteristics of the included studies are summarized in Table 1.

**Table 1:** Basic information of literature characteristics.

Study	Year of publication	Country	Study regions	Diseases	Study design	Factors	Group	Sample (total/case/control)	Age of Cases	Age of Controls	Gender of cases(F/M)	Gender of Control(F/M)	Duration of study(year)	OR, RR, HR, RH(95% CI)	Outcomes
Saydah 2010I	2010	USA	USA	Diabetes	Cross-sectional	Educational level	<High school vs. College degree	224,718/110242/114476	na	45.4(0.04)	57,326/52916	53,575/60901	11	2.46(2.15,2.82)	Diabetes-related mortality
Saydah 2010II	2010	USA	USA	Diabetes	Cross-sectional	Family income	<100% vs. ≥ 400% percent FPL	199,211/62092/137119	48.9(0.07)	46.6(0.05)	38,808/23284	65,543/71576	11	2.94(2.53,3.42)	Diabetes-related mortality
Secrest 2011I	2011	USA	Pittsburgh	T1D	Cohort	Education	without vs. with a college degree	34/3/0	28.9 ± 1.6		15/19		13.9	3.0(1.2,7.8)	Mortality
Secrest 2011II	2011	USA	Pittsburgh	T1D	Cohort	Income	Lowest vs. highest	34/5/2	28.9 ± 1.6		15/19		13.9	3.2(0.8,13.5)	Mortality
Secrest 2011III	2011	USA	Pittsburgh	T1D	Cohort	Occupation	Lowest vs. highest	34/12/5	28.9 ± 1.6		15/19		13.9	1.6(0.8,3.2)	Mortality
Vandenheede 2011I_M	2011	Belgium	Belgian/North African	Diabetes	Cohort	Education	Pre-primary vs. Tertiary	73,266/10247/63019	25–74		0/10247	63,019/0	5	2.55(1.68,3.88)	Diabetes-related mortality
Vandenheede 2011II_F	2011	Belgium	Belgian/North African	Diabetes	Cohort	Education	Pre-primary vs. Tertiary	78,302/12790/65512	25–74		12,790/0	0/65512	5	7.51(3.53,15.95)	Diabetes-related mortality
Vandenheede 2011III_M	2011	Belgium	Belgian/North African	Diabetes	Cohort	Housing status	Low-quality tenant vs. High-quality owner	75,102/36115/38987	25–74		0/36115	38,987/0	5	1.68(1.15,2.45)	Diabetes-related mortality
Vandenheede 2011IV_F	2011	Belgium	Belgian/North African	Diabetes	Cohort	Housing status	Low-quality tenant vs. High-quality owner	76,193/32512/43681	25–74		32,512/0	0/43681	5	5.08(2.90,8.89)	Diabetes-related mortality
Vandenheede 2013I_M	2013	UK	Flanders	Diabetes	Cohort	Own education	Lower secondary vs. Higher education	35–54 years:1919590PY/165/56; 55–74 years:1913651PY/1892/254	35–74		2057	310	10	1.38(1.20,1.60)	ASMRs
Vandenheede 2013II_F	2013	UK	Flanders	Diabetes	Cohort	Own education	Lower secondary vs. Higher education		35–74				10	2.18(1.22,3.89)	ASMRs
Vandenheede 2013III_M	2013	UK	Flanders	Diabetes	Cohort	Partner’s education	Lower secondary vs. Higher education	35–54 years:1779615PY/171/52; 55–74 years:2129910PY/2040/165	35–74		2,211	217	10	1.22(1.04,1.44)	ASMRs
Vandenheede 2013IV_F	2013	UK	Flanders	Diabetes	Cohort	Partner’s education	Lower secondary vs. Higher education		35–74				10	1.95(1.53,2.48)	ASMRs
Vandenheede 2013V_M	2013	UK	Flanders	Diabetes	Cohort	Housing status	Low-quality tenant vs. High-quality owner	35–54 years:253532PY/37/86; 55–74 years:155752PY/260/652	35–74		297	738	10	2.71(1.68,4.37)	ASMRs
Vandenheede 2013VI_F	2013	UK	Flanders	Diabetes	Cohort	Housing status	Low-quality tenant vs. High-quality owner		35–74				10	2.56(2.17,3.03)	ASMRs
Dalsgaard 2015I_M	2015	Denmark	Danish	Type 2 diabetes	Cohort	Educational level	≤10 years vs. > 15 years	139,681/62059/16541	55.2 ± 8.5		0/na	0/na	0–2, 2–4, 4–6, >6	1.21(1.12,1.31)	All-cause mortality
Dalsgaard 2015II_F	2015	Denmark	Danish	Type 2 diabetes	Cohort	Educational level	≤10 years vs. > 15 years				na/0	na/0	0–2, 2–4, 4–6, >6	1.29(1.17,1.42)	All-cause mortality
Dalsgaard 2015III_M	2015	Denmark	Danish	Type 2 diabetes	Cohort	Income level	20 vs. 80 percentile	139,681/33850/21419			0/na	0/na	0–2, 2–4, 4–6, >6	1.42(1.30,1.56)	All-cause mortality
Dalsgaard 2015IV_F	2015	Denmark	Danish	Type 2 diabetes	Cohort	Income level	20 vs. 80 percentile				na/0	na/0	0–2, 2–4, 4–6, >6	1.40(1.26,1.56)	All-cause mortality
Kim 2016	2016	Korea	Korea	diabetes mellitus	Cohort	Socioeconomic status	Lowest 30% vs. highest 30%	20,220/4903/8302	56.1 ± 11.4	53.8 ± 11.7	2972/5330	2393/2510	7.9	1.31(1.10,1.55)	mortality
Rawshani 2016I	2016	Sweden	Sweden	Type 2 diabetes	Cohort	Income quintile	Lowest vs. highest	115,029/23734/26942	59.0 ± 10.7	57.3 ± 8.1	24,567/17141	9361/38891	10	2.12(2.01,2.24)	Mortality
Rawshani 2016II	2016	Sweden	Sweden	Type 2 diabetes	Cohort	Educational level	<9 years vs. college/university	87,781/29525/21151	60.4 ± 8.6	57.2 ± 9.2	30,117/45745	14,907/24322	10	1.23(1.18,1.30)	Mortality
Shin 2016I_M	2016	Korea	Korea	Type 2 diabetes	Cohort	Income	Low vs. high	6156/2016/2264	44–75	44–75	0/2016	2264/0	7	1.56(1.18,2.05)	Mortality
Shin 2016II_F	2016	Korea	Korea	Type 2 diabetes	Cohort	Income	Low vs. high	7705/2164/2391			2164/0	0/2391	7	1.61(1.16,2.23)	Mortality
Shin 2016III_M	2016	Korea	Korea	Type 2 diabetes	Cohort	Job status	Unemployed vs. maintain Job	7156/2793/3019			0/2793	3019/0	7	3.78(2.81,5.09)	Mortality
Shin 2016IV_F	2016	Korea	Korea	Type 2 diabetes	Cohort	Job status	Unemployed vs. maintain Job	7705/6049/963			6049/0	0/963	7	9.78(2.39,39.98)	Mortality
Shin 2016V_M	2016	Korea	Korea	Type 2 diabetes	Cohort	Residence	Rural areas vs. Seoul	7156/1528/805			0/1528	805/0	7	1.11(0.86,1.45)	Mortality
Shin 2016VI_F	2016	Korea	Korea	Type 2 diabetes	Cohort	Residence	Rural areas vs. Seoul	7705/1605/880			1605/0	0/880	7	1.30(0.91,1.85)	Mortality
Aguilar-Palacio 2017I_M	2017	Spain	Spanish	Diabetes	Cohort	Income	Deprivation vs. affluent census tracts	na	16–29		na	na	10	1.08(0.59,1.98)	Mortality
Aguilar-Palacio 2017II_F	2017	Spain	Spanish	Diabetes	Cohort	INCOME	Deprivation vs. affluent census tracts	na	16–29		na	na	10	0.62(0.27,1.41)	Mortality
Blomster 2017	2017	Australia	Australia	Type 2 diabetes	RCTs	Education	Low vs. higher education	11,140/4024/7116	67.1 ± 6.3	65.0 ± 6.3	2748/1276	1985/5131	5	1.34(1.18,1.52)	Mortality
Shin 2018I_M	2018	Korea	Korea	Diabetes	Cohort	Income	Low vs. high	34,403/12813/21590	56(48–64)		12,813/0	0/21590	12	1.32(1.23,1.41)	Mortality
Shin 2018II_F	2018	Korea	Korea	Diabetes	Cohort	Income	Low vs. high	21,036/9854/11182			0/9854	11,182/0	12	1.21(1.10,1.33)	Mortality
Shulman 2018	2018	Canada	Ontario	Type 1 diabetes	Cohort	Income	Most vs. least deprived	na	<19		na	na	6.6 (3.0–10.8) and 6.5 (3.0–10.7)	2.03(1.13,3.63)	Mortality
Campbell 2020I_M	2020	UK	Scotland	Type 1 diabetes	Cohort	SIMD	20% most vs. least deprived areas	274,095/55217/50412	0–39, 40–59, 60–79 and ≥80 years		120,527/81368		10	1.21(0.98,1.49)	Mortality
Campbell 2020II_F	2020	UK	Scotland	Type 1 diabetes	Cohort	SIMD	20% most vs. least deprived areas						10	1.55(1.20,1.99)	Mortality
Chen 2022I_M	2022	China	Changshu and Huaian	Type 2 diabetes	Cohort	SES	Low vs. high	6971/2098/2651	na	na	0/2098	0/2651	5.7 ± 0.9	1.87(1.55,2.25)	All-cause mortality
Chen 2022II_F	2022	China	Changshu and Huaian	Type 2 diabetes	Cohort	SES	Low vs. high	10,582/6158/2219	na	na	6158/0	2219/0		1.53(1.26,1.86)	All-cause mortality
Lusk 2022	2022	Durham	Durham	Type 1 and 2 Diabetes	Cohort	ADI	ADI 86-100(low SES) vs. ADI 1-15(high SES)	130,100/19300/19800	>65	>65	66,507/63644		3	1.22(1.11,1.34)	30-day mortality
Lee 2023	2023	Korea	Korea	Type 2 diabetes	Cohort	Income	Lowest vs. highest income status	1,943,354/401061/594593	na	na	na	na	5	0.89(0.80,0.98)	All-cause mortality
Liao 2023	2023	China	Taiwan	Type 2 diabetes	Cohort	Educational level	Elementary or below vs. College or above	1,307,963/667419/118596	na	na	620,798/687165		16	1.89(1.85,1.92)	P4P participation on mortality
Rosella 2023	2023	Canada	Ontario	Diabetes	Cohort	Income	20% lowest vs. highest income	1,741,098/409403/277957	58.06 ± 14.76		204,996/204407	122,579/155378	25	1.05(1.04,1.06)	All-cause mortality
Li 2025	2025	China	US	Diabetes	Cohort	Household income	Low vs. high household income	2069/477/425	na	na	na	na	18	2.78(1.89,4.17)	All-cause mortality

### 3.2 Assessment of study quality

**Figure 2:**
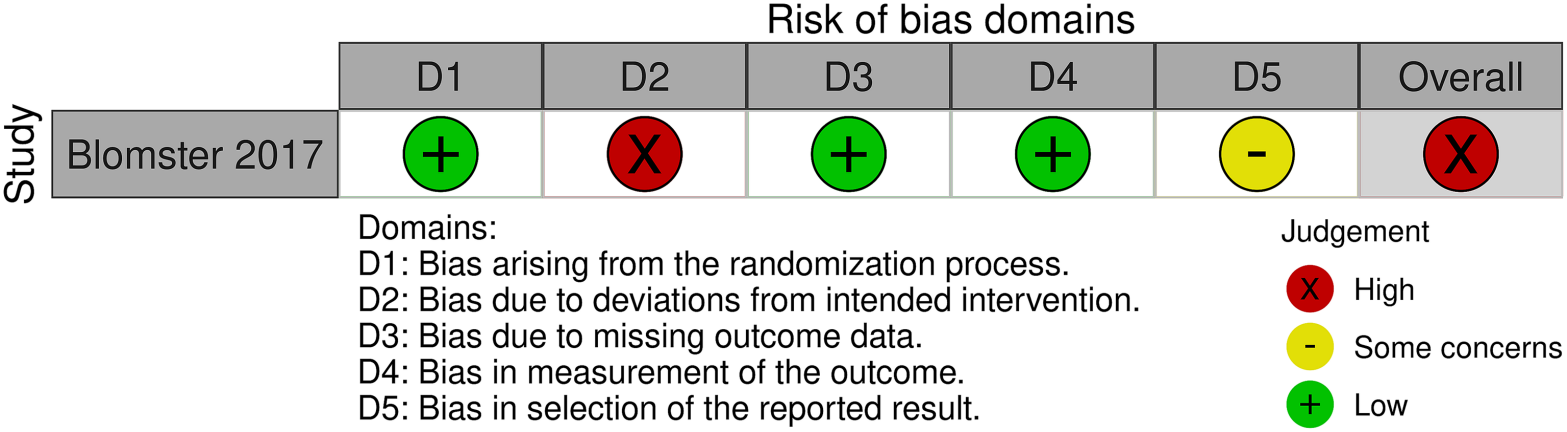
Risk of bias assessment for the randomized controlled trial using the RoB 2 tool.

**Figure 3:**
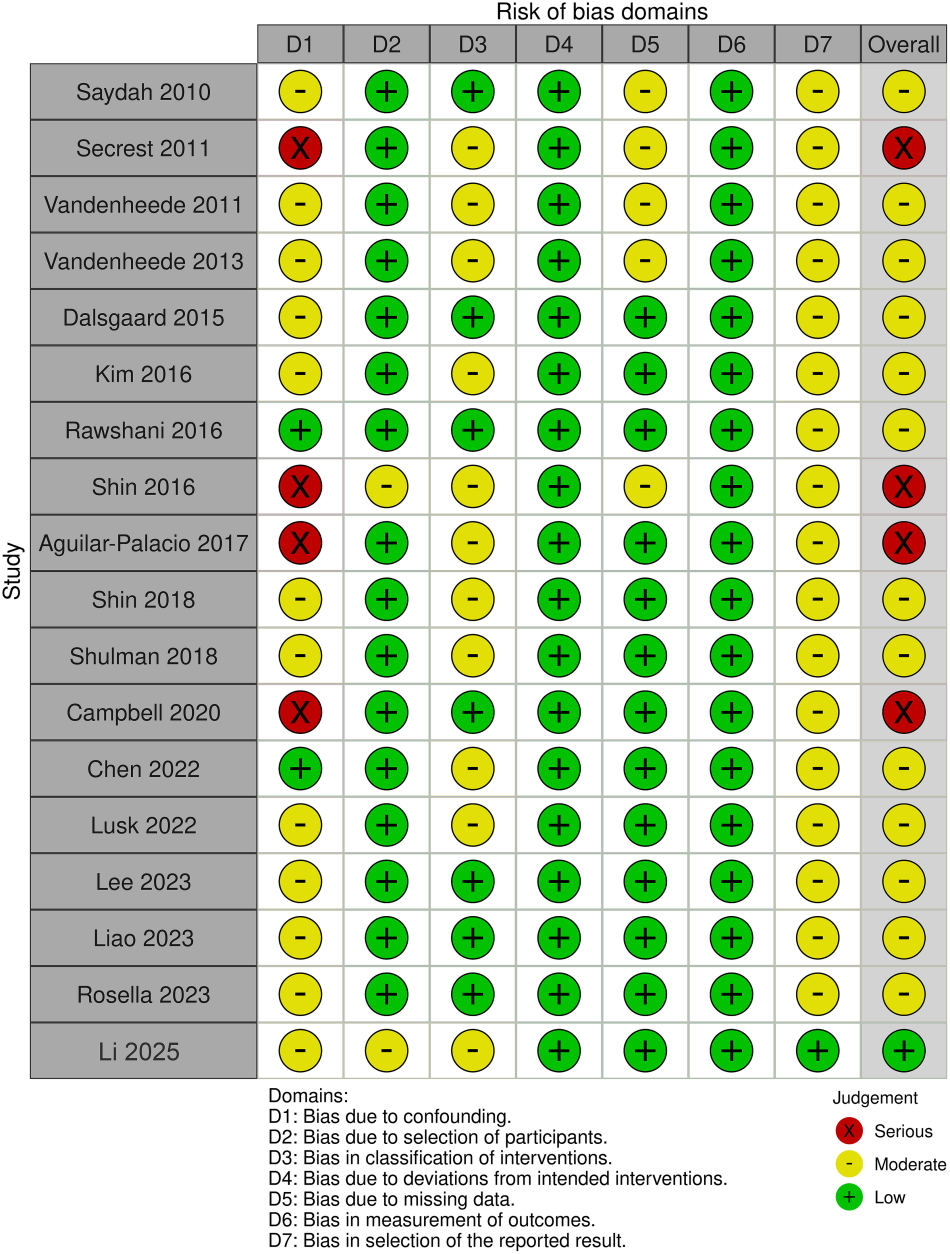
Risk of bias assessment for non-randomized studies using the ROBINS-I tool.

Quality assessment revealed that among the 19 included studies, 1 was rated as low risk of bias, 13 as moderate risk, and 5 as high risk overall. Among 18 non-randomized studies assessed with ROBINS-I, 4 showed serious risk of bias, mainly due to confounding (19, 23, 28, 32). One study (37) was judged as low risk of bias, while the remaining studies were classified as moderate risk. The single randomized controlled trial (29), assessed using the RoB 2 tool, was rated as high risk of bias, mainly due to deviations from the intended intervention. Because only one RCT was included, and the vast majority of studies were observational, the RCT did not materially influence the pooled effect. Detailed risk-of-bias assessments are presented in Figures 2, 3.

### 3.3 Results of meta-analysis

**Figure 4:**
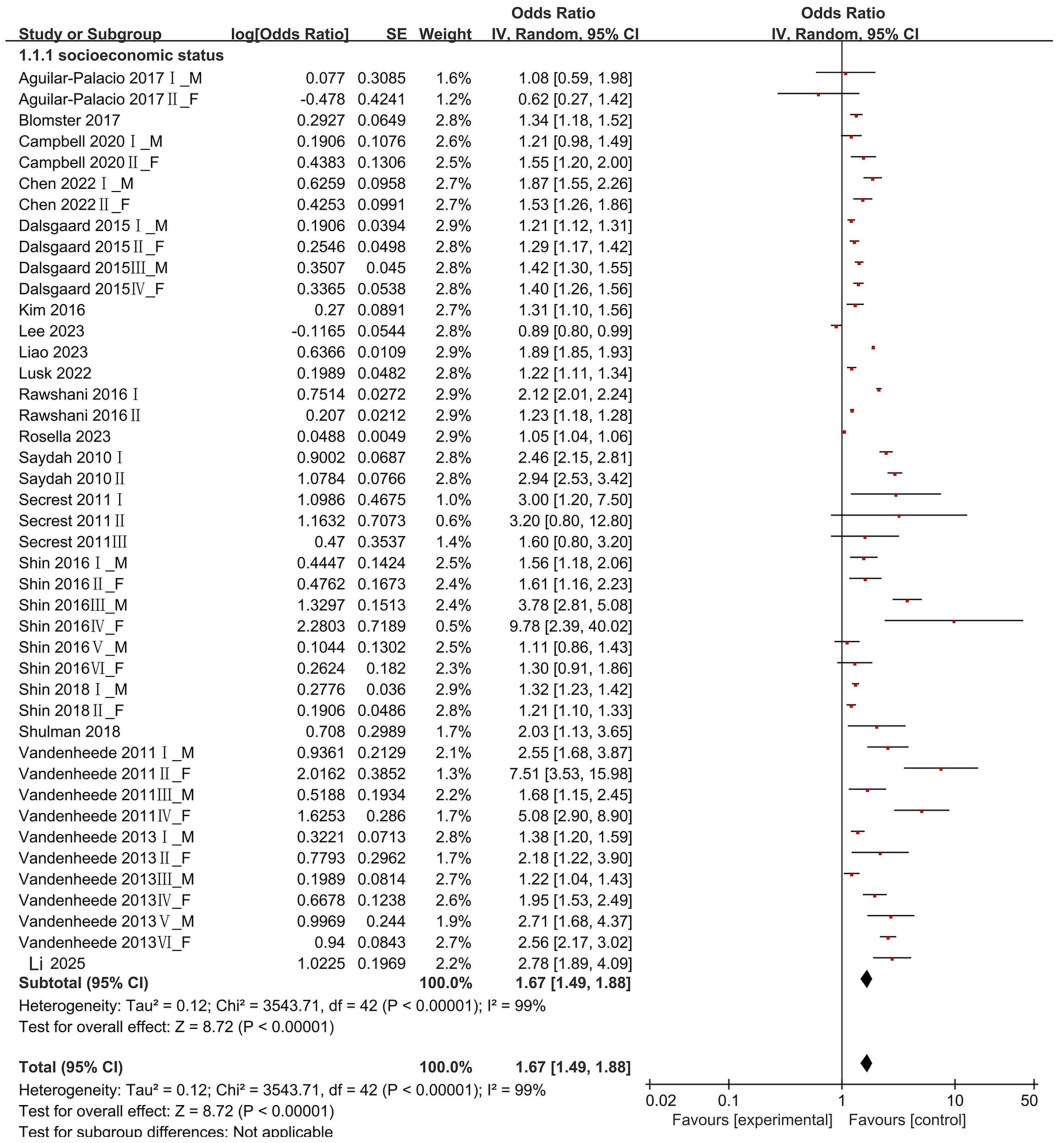
Forest plot of the association between low socioeconomic status and all-cause mortality in patients with diabetes.

The meta-analysis of 19 studies demonstrated that low socioeconomic status (SES) was significantly associated with higher all-cause mortality among patients with diabetes (OR = 1.67; 95% CI: 1.49–1.88; *p* < 0.00001). The association was consistently observed across all included studies, with no evidence of protective effects. However, heterogeneity between studies was substantial (*I*^2^ = 99%), which may reflect differences in SES measures (e.g., education, income), study populations, analytical approaches, as well as other unmeasured factors. Despite the high heterogeneity, an association between socioeconomic disadvantage and mortality risk in diabetes was observed across studies. Detailed results are shown in Figure 4.

#### 3.3.1 Income

By country: Significant differences were observed (*p* < 0.0001). Low income was associated with increased mortality risk in most populations, with the strongest effects in China (OR = 2.18; 95% CI: 1.51–3.14) and Sweden (OR = 2.12; 95% CI: 2.01–2.24). Significant but smaller associations were observed in Denmark and the United Kingdom, whereas no significant association was found in Spain (OR = 0.88; 95% CI: 0.52–1.49). The pooled effect confirmed an elevated mortality risk among low-income groups (OR = 1.49; 95% CI: 1.24–1.79; *I*^2^ = 99%). Detailed results are shown in Supplementary Figure S1A.

By gender: Low income increased mortality risk in both sexes, with comparable effect sizes: men (OR = 1.35; 95% CI: 1.28–1.43; *I*^2^ = 6%) and women (OR = 1.31; 95% CI: 1.23–1.40; *I*^2^ = 62%). The difference between sexes was not statistically significant (*p* = 0.50). Detailed results are shown in Supplementary Figure S1B.

By study design: Cohort studies demonstrated an overall effect of OR = 1.63 (95% CI 1.36–1.95; *I*^2^ = 98%), while the single cross-sectional study showed a stronger effect (OR = 2.46; 95% CI 2.15–2.81; *p* = 0.0003). Given the limited number of cross-sectional studies, this difference should be interpreted with caution. Detailed results are presented in Supplementary Figure S1C.

#### 3.3.2 Education

By country: Low educational attainment was associated with increased mortality across most countries, particularly in Belgium (OR = 4.17; 95% CI: 1.45–11.97) and the USA (OR = 3.00; 95% CI: 1.20–7.50). Moderate but significant effects were also observed in Australia, Denmark, Sweden, and the UK. Detailed results are presented in Supplementary Figure S3A.

By gender: Lower education increased mortality risk in both men (OR = 1.37; 95% CI 1.16–1.61; *I*^2^ = 78%) and women (OR = 2.24; 95% CI 1.37–3.64; *I*^2^ = 90%). The gender difference was not statistically significant (*p* = 0.06) but suggested a potentially stronger effect among women. Detailed results are presented in Supplementary Figure S3B.

By study design: Cohort studies demonstrated an overall effect of OR = 1.63 (95% CI 1.36–1.95; *I*^2^ = 98%), while the cross-sectional study indicated a stronger effect (OR = 2.46; 95% CI 2.15–2.81; *p* = 0.0003). As only one cross-sectional study was available, results should be interpreted with caution. Detailed results are presented in Supplementary Figure S3C.

#### 3.3.3 Housing status

Disadvantaged housing conditions were associated with a significantly higher risk of all-cause mortality (OR = 1.99; 95% CI 1.77–2.24; *I*^2^ = 89%). By country, the effect was strongest in Belgium (OR = 2.38) and the UK (OR = 2.58), and not statistically significant in Korea (OR = 1.17). By gender, the effect was significant in both men (OR = 1.66; 95% CI 1.01–2.74) and women (OR = 2.48; 95% CI 1.36–4.51), with no significant sex difference (*p* = 0.31). Detailed results are shown in Supplementary Figures S5A,B.

#### 3.3.4 Employment status

Unemployment was associated with a substantially higher mortality risk (OR = 3.33; 95% CI: 1.55–7.17; *I*^2^ = 72%). Country-specific effects varied: no significant association was found in the United States (OR = 1.60; 95% CI: 0.80–3.20), whereas a strong effect was observed in Korea (OR = 4.69; 95% CI: 2.15–10.23; *p* = 0.0001). The between-country difference was statistically significant (*p* = 0.04). Detailed results are presented in Supplementary Figure S7.

### 3.4 Publication bias

Egger’s test indicated no significant publication bias for most analyses (*p* > 0.25), except for education by gender (*p* = 0.002). Funnel plots showed mild asymmetry in some subgroups, which likely resulted from heterogeneity rather than true publication bias. However, these findings should be interpreted with caution because some subgroup analyses included only a small number of studies. Funnel plots and detailed statistical results are provided in Supplementary Figures S2–S8.

### 3.5 Sensitivity analysis

The results remained stable in leave-one-out sensitivity analyses, as exclusion of individual studies did not materially affect the pooled estimates for income or education (Supplementary Figures S9–S11).

## 4 Discussion

The results of our meta-analysis indicate that low SES is associated with a 67% higher risk of all-cause mortality among individuals with diabetes (OR = 1.67; 95% CI: 1.49–1.88). All of the studies showed the same direction of effect, with none showing a protective association. In subsequent analyses, subgroup analyses suggested that different SES components may contribute unequally to mortality risk, with the strongest association observed for employment status (OR = 3.33). Housing conditions (OR = 1.99) and educational attainment (OR ≈ 1.63), followed by income (OR = 1.49), also showed significant positive associations. This indicates that multiple dimensions of socioeconomic disadvantage may influence survival, although their relative contributions appear to differ.

One possible explanation for this phenomenon is unequal access to healthcare. Effective diabetes management requires regular monitoring, timely treatment, and consistent access to medications and supplies. However, these resources are often more difficult to obtain for socioeconomically disadvantaged patients. Limited insurance coverage (38–42) and high healthcare costs (10, 32) may contribute to delayed diagnosis, poorer glycemic control, and reduced treatment adherence. This may partly explain our findings regarding employment status, which showed the strongest association with mortality in our analysis (OR = 3.33). This may be because employment status influences both access to insurance coverage and the ability to afford healthcare-related expenses. Similarly, lower income can further restrict access to healthcare resources and the affordability of treatment. In our analysis, income was also associated with increased mortality risk (OR = 1.49). This is in line with previous studies showing an inverse relationship between income level and mortality risk among individuals with diabetes (17, 33, 36), a pattern that persists even in countries with broadly universal healthcare coverage, such as Denmark and the UK (15, 25). Another potential explanation for higher mortality may be differences in the use of specialist diabetes care. Evidence suggests that individuals with lower SES are less likely to achieve optimal disease control, partly due to lower adherence to treatment and reduced follow-up care (43). This is reflected in less frequent attendance at diabetes centers, lower treatment intensity, and fewer therapy adjustments (44). Finally, differences between individuals with higher and lower SES are also reflected in a higher risk of diabetes-related complications among those in more disadvantaged situations. These include retinopathy, cardiovascular complications, and diabetic ketoacidosis (45, 46).

However, the problem of increased mortality among individuals in disadvantaged socioeconomic conditions extends beyond access to medical care alone. Housing-related deprivation has been associated with poorer glycemic control, while unstable living conditions can make self-management, medication adherence, and healthy eating more difficult (47, 48). This pattern is consistent with our analysis, in which housing conditions demonstrated a strong association with mortality risk (OR = 1.99). In addition, individuals with lower SES often face restricted access to healthy food and transportation (49), which may further complicate disease management. Educational attainment represents another important dimension of socioeconomic disadvantage, which was also reflected in the results of our meta-analysis. Lower education and limited health literacy may reduce patients’ capacity for self-management, treatment adherence, and participation in preventive care (10). Furthermore, evidence from systematic reviews indicates that lower health literacy is associated with poorer glycemic control (50). New technologies that have the potential to support diabetes management are also less accessible to individuals with lower SES due to disparities in digital literacy, internet access, and financial constraints (51). In our analysis, the association between education and mortality appeared stronger among women, suggesting that educational inequalities may disproportionately affect disease management in female populations.

Behavioral patterns that may influence mortality in individuals with diabetes are also linked to patients’ SES, and their effects may accumulate. For instance, those living in more deprived conditions are more likely to engage in unhealthy behaviors, including smoking, physical inactivity, poor diet, and prolonged sedentary time, which may contribute to differences in diabetes management and outcomes (15). In addition, chronic psychosocial stress, which is more common among lower-SES groups, may further reinforce unhealthy behaviors, including tobacco use, reduced physical activity, and poor dietary patterns (52). On the other hand, studies have shown that the protective effects of healthy lifestyle factors, such as non-smoking, regular physical activity, and a balanced diet, appear to be stronger among individuals with higher SES. This may indicate that adverse socioeconomic conditions can limit the beneficial effects of such behaviors (53).

Importantly, the relationship between type 2 diabetes and socioeconomic status appears to be bidirectional. While lower SES increases the risk of developing diabetes, living with the disease may itself contribute to further socioeconomic disadvantage. Patients living with diabetes are exposed to increased healthcare expenditures and significant out-of-pocket costs. In addition, diabetes negatively affects productivity, contributes to higher rates of unemployment, and is associated with decreased educational attainment, all of which may adversely influence long-term SES. Taken together, these interconnected pathways may create a self-reinforcing cycle of disadvantage, making both disease management and social mobility increasingly difficult (54).

Considering these mechanisms, socioeconomic disadvantage likely reflects a combination of interrelated conditions rather than isolated exposures. Therefore, composite indicators such as SES may be helpful in guiding strategies aimed at addressing overlapping determinants and improving survival outcomes in individuals with diabetes. Importantly, these findings should also be interpreted in the context of health inequalities. Low socioeconomic status may reflect social and economic barriers affecting access to healthcare, preventive services, and long-term diabetes management. In this context, reducing mortality among individuals with diabetes may require not only optimized clinical treatment but also public health strategies aimed at reducing socioeconomic disparities.

However, several limitations should be considered when interpreting these findings. First, most included studies were observational, which limits causal inference. The inclusion of a single RCT among predominantly observational evidence may also have contributed to methodological heterogeneity. Second, substantial heterogeneity was observed between studies (I^2^ = 99%), likely reflecting differences in SES definitions, study populations, and analytical approaches. SES indicators varied across studies—spanning income, education, occupation, and area-level deprivation—and composite versus domain-specific measures were not consistently reported. As a result, the pooled estimate should be interpreted as a summary of the broadly adverse association observed across settings rather than a precise effect size. Third, reporting of key determinants—such as age, lifestyle factors, multimorbidity, and access to healthcare—differed to some extent between studies, which increases the likelihood of residual confounding. Reverse causality is also a relevant issue, as more advanced illness may contribute to declines in socioeconomic status. Finally, the use of all-cause mortality as the primary outcome limits the ability to distinguish diabetes-related deaths from those unrelated to diabetes-specific pathways. Future research should incorporate diabetes-attributed or cause-specific mortality, which may offer a clearer understanding of the mechanisms linking socioeconomic status with mortality among individuals with diabetes.

## 5 Conclusion

Lower socioeconomic status is significantly associated with increased all-cause mortality among individuals with diabetes. Higher mortality risk was consistently linked to lower income, education, occupational status, and housing conditions. These findings suggest that survival outcomes in diabetes may depend not only on biological and clinical factors but also on broader socioeconomic conditions. Addressing socioeconomic disadvantage and reducing structural barriers to healthcare may therefore help improve survival in this population.

## Data Availability

The original contributions presented in the study are included in the article/Supplementary material, further inquiries can be directed to the corresponding author.

## Footnotes

1
https://mcguinlu.shinyapps.io/robvis/

## References

[1] IDF Diabetes Atlas. 11th ed. Brussels: International Diabetes Federation; (2025). Available online at: https://diabetesatlas.org/ (Accessed June 06, 2026).

[2] Emerging Risk Factors Collaboration. Diabetes mellitus, fasting glucose, and risk of cause-specific death. N Engl J Med (2011);364:829–841. doi: 10.1056/NEJMoa1008862 21366474 PMC4109980

[3] Raghavan S Vassy JL Ho Y Song RJ Gagnon DR Cho K . Diabetes mellitus–related all-cause and cardiovascular mortality in a national cohort of adults. J Am Heart Assoc. (2019) 8:e011295. doi: 10.1161/jaha.118.011295, 30776949 PMC6405678

[4] Li S Wang J Zhang B Li X Liu Y. Diabetes mellitus and cause-specific mortality: a population-based study. Diabetes Metab J. (2019) 43:319–41. doi: 10.4093/dmj.2018.0060, 31210036 PMC6581547

[5] Flood D Zhang YS Nichols E Li C Zaninotto P Langa KM . Diabetes and all-cause mortality among middle-aged and older adults in China, England, Mexico, rural South Africa, and the USA. BMJ Open Diabetes Res Care. (2025) 13:e004678. doi: 10.1136/bmjdrc-2024-004678 PMC11931896 40101978

[6] World Health Organization. Diabetes. Geneva: World Health Organization; (2023). Available online at: https://www.who.int/news-room/fact-sheets/detail/diabetes (Accessed June 06, 2026).

[7] GBD 2021 Diabetes Collaborators. Global, regional, and national burden of diabetes from 1990 to 2021 and projections to 2050: a systematic analysis for the global burden of disease study 2021. Lancet. (2023) 402:203–34. doi: 10.1016/S0140-6736(23)01301-6 37356446 PMC10364581

[8] Ferede YM Erlandsson K Gebrie MH Beshah DT Mohammed OY Azagew AW . Global prevalence of multimorbidity among people living with type 2 diabetes: a systematic review and meta-analysis. BMC Public Health. (2025) 25:4137. doi: 10.1186/s12889-025-25570-3 41382017 PMC12802173

[9] Social Determinants of Health. Geneva: World Health Organization; (2023). Available online at: https://www.who.int/health-topics/social-determinants-of-health (Accessed June 06, 2026).

[10] Hill-Briggs F Adler NE Berkowitz SA Chin MH Gary-Webb TL Navas-Acien A . Social determinants of health and diabetes: a scientific review. Diabetes Care. (2020) 44:258–79. doi: 10.2337/dci20-0053 33139407 PMC7783927

[11] Kyrou I Tsigos C Mavrogianni C Cardon G Van Stappen V Latomme J . Sociodemographic and lifestyle-related risk factors for identifying vulnerable groups for type 2 diabetes: a narrative review with emphasis on data from Europe. BMC Endocr Disord. (2020) 20:134. doi: 10.1186/s12902-019-0463-3, 32164656 PMC7066728

[12] Deng Y Moniruzzaman M Rogers B Hu L Jagannathan R Tamura K. Racial, ethnic, and socioeconomic disparities in diabetes: findings from NHANES 2007–2020. Prev Med Rep. (2025) 50:102957. doi: 10.1016/j.pmedr.2024.102957 40007950 PMC11852695

[13] Agardh E Allebeck P Hallqvist J Moradi T Sidorchuk A. Type 2 diabetes incidence and socio-economic position: a systematic review and meta-analysis. Int J Epidemiol. (2011) 40:804–18. doi: 10.1093/ije/dyr029, 21335614

[14] Meisters R Koster A Albers J Sezer B van Greevenbroek MMJ de Galan BE . Early life socioeconomic inequalities and type 2 diabetes incidence. Diabetes Res Clin Pract. (2024) 217:111855. doi: 10.1016/j.diabres.2024.111855 39265827

[15] Li J Li J Fu Y Zhang K Tan X Wang N . Socioeconomic deprivation, unhealthy lifestyle, and premature mortality in type 2 diabetes. Am J Med. (2025) 138:1248–56.e7. doi: 10.1016/j.amjmed.2025.05.001 40334765

[16] Walker JJ Livingstone SJ Colhoun HM Lindsay RS McKnight JA Morris AD . Effect of socioeconomic status on mortality among people with type 2 diabetes. Diabetes Care. (2011) 34:1127–32. doi: 10.2337/dc10-1862, 21421800 PMC3114515

[17] Lee HS Park JC Chung I Liu J Lee SS Han K. Sustained low income, income changes, and risk of all-cause mortality in individuals with type 2 diabetes. Diabetes Care. (2023) 46:92–100. doi: 10.2337/dc21-2305, 36367896

[18] Komura T Kondo N Bhatt K Inoue K. Association between educational status and mortality according to diabetes status among US adults. Mayo Clin Proc Innov Qual Outcomes. (2023) 7:203–11. doi: 10.1016/j.mayocpiqo.2023.04.007, 37304061 PMC10250573

[19] Shin D Kim JM Tandi TE Park EC. Impact of change in job status on mortality in newly diagnosed type 2 diabetes: a 7-year cohort study. Diabetes Metab Syndr. (2016) 10:S1–6. doi: 10.1016/j.dsx.2015.08.012, 26341929

[20] Vandenheede H Vanroelen C Gadeyne S De Grande H Deboosere P. Household-based socioeconomic position and diabetes-related mortality among married and cohabiting persons. J Epidemiol Community Health. (2013) 67:765–71. doi: 10.1136/jech-2012-202290 23761411

[21] Page MJ McKenzie JE Bossuyt PM Boutron I Hoffmann TC Mulrow CD . The PRISMA 2020 statement. BMJ. (2021) 372:n71. doi: 10.1136/bmj.n71 33782057 PMC8005924

[22] Saydah S Lochner K. Socioeconomic status and risk of diabetes-related mortality in the U.S. Public Health Rep. (2010) 125:377–88. doi: 10.1177/003335491012500306, 20433032 PMC2848262

[23] Secrest AM Costacou T Gutelius B Miller RG Songer TJ Orchard TJ. Association of socioeconomic status with mortality in type 1 diabetes: the Pittsburgh epidemiology of diabetes complications study. Ann Epidemiol. (2011) 21:367–73. doi: 10.1016/j.annepidem.2011.02.011, 21458730 PMC3070912

[24] Vandenheede H Lammens L Deboosere P Gadeyne S De Spiegelaere M. Ethnic differences in diabetes-related mortality in the Brussels-capital region (2001–2005): the role of socioeconomic position. Int J Public Health. (2011) 56:533–9. doi: 10.1007/s00038-011-0235-y, 21302129

[25] Dalsgaard EM Skriver MV Sandbaek A Vestergaard M. Socioeconomic position, type 2 diabetes and long-term risk of death. PLoS One. (2015) 10:e0124829. doi: 10.1371/journal.pone.0124829, 25942435 PMC4420496

[26] Kim NH Kim TJ Kim NH Choi KM Baik SH Choi DS . Relative and combined effects of socioeconomic status and diabetes on mortality: a nationwide cohort study. Medicine (Baltimore). (2016) 95:e4403. doi: 10.1097/MD.0000000000004403, 27472736 PMC5265873

[27] Rawshani A Svensson AM Zethelius B Eliasson B Rosengren A Gudbjörnsdottir S. Association between socioeconomic status and mortality, cardiovascular disease, and cancer in patients with type 2 diabetes. JAMA Intern Med. (2016) 176:1146–54. doi: 10.1001/jamainternmed.2016.2940, 27367969

[28] Aguilar-Palacio I Martinez-Beneito MA Rabanaque MJ Borrell C Cirera L Daponte A . Diabetes mellitus mortality in Spanish cities: trends and geographical inequalities. Prim Care Diabetes. (2017) 11:453–60. doi: 10.1016/j.pcd.2017.05.006 28623082

[29] Blomster JI Zoungas S Woodward M Neal B Harrap S Poulter N . The impact of level of education on vascular events and mortality in patients with type 2 diabetes mellitus: results from the ADVANCE study. Diabetes Res Clin Pract. (2017) 127:212–7. doi: 10.1016/j.diabres.2017.03.015, 28395214

[30] Shin WY Kim HC Lee T Jeon DH Ha KH Kim DJ . Combined effects of diabetes and low household income on mortality: a 12-year follow-up study of 505,677 Korean adults. Diabet Med. (2018) 35:1345–54. doi: 10.1111/dme.13695, 29851428

[31] Shulman R Luo J Shah BR. Mental health visits and low socio-economic status in adolescence are associated with complications of type 1 diabetes in early adulthood: a population-based cohort study. Diabet Med. (2018) 35:920–8. doi: 10.1111/dme.13633, 29608218

[32] Campbell RAS Colhoun HM Kennon B McCrimmon RJ Sattar N McKnight J . Socioeconomic status and mortality in people with type 1 diabetes in Scotland, 2006–2015: a retrospective cohort study. Diabet Med. (2020) 37:2081–8. doi: 10.1111/dme.14239 31967666

[33] Chen YJ Su J Qin Y Shen C Pan EC Yu H . A prospective cohort study on socioeconomic status and risk of all-cause mortality among patients with type 2 diabetes based on latent class analysis. Zhonghua Liu Xing Bing Xue Za Zhi. (2022) 43:1619–25. Chinese. doi: 10.3760/cma.j.cn112338-20220107-00010, 36456494

[34] Lusk JB Hoffman MN Clark AG Bae J Corsino L Hammill BG. Neighborhood socioeconomic deprivation and 30-day mortality and readmission for patients admitted for diabetes management. Diabetes Care. (2022) 45:e169–70. doi: 10.2337/dc22-0913, 36107404

[35] Liao YS Tsai WC Chiu LT Kung PT. Educational attainment affects diagnostic time in type 2 diabetes mellitus and mortality risk among participants in a diabetes pay-for-performance program. Health Policy. (2023) 138:104917. doi: 10.1016/j.healthpol.2023.104917 37776765

[36] Rosella LC Kornas K Negatu E Zhou L. Variations in all-cause mortality, premature mortality and cause-specific mortality among persons with diabetes in Ontario, Canada. BMJ Open Diabetes Res Care. (2023) 11:e003378. doi: 10.1136/bmjdrc-2023-003378, 37130629 PMC10163552

[37] Li Z Zhou L Wu Y Ding T Gan Y Fan X. Associations of healthy lifestyle and family income-to-poverty ratio with all-cause mortality among people with prediabetes and diabetes: a prospective cohort study. BMC Public Health. (2025) 25:24. doi: 10.1186/s12889-024-21206-0, 39754064 PMC11697864

[38] Kazemian P Shebl FM McCann N Walensky RP Wexler DJ. Evaluation of the cascade of diabetes care in the United States, 2005–2016. JAMA Intern Med. (2019) 179:1376–85. doi: 10.1001/jamainternmed.2019.2396, 31403657 PMC6692836

[39] Danaei G Friedman AB Oza S Murray CJ Ezzati M. Diabetes prevalence and diagnosis in US states: analysis of health surveys. Popul Health Metrics. (2009) 7:16. doi: 10.1186/1478-7954-7-16, 19781056 PMC2764564

[40] American Diabetes Association. Economic costs of diabetes in the U.S. in 2017. Diabetes Care. (2018) 41:917–28. doi: 10.2337/dci18-0007, 29567642 PMC5911784

[41] Almadfaa RO. Racial and socioeconomic disparities in the utilization of diabetes medication: a comprehensive analysis of NHANES 2011–2023. Res Social Adm Pharm. (2025) 22:326–32. doi: 10.1016/j.sapharm.2025.10.013, 41177698

[42] Ngo-Metzger Q Sorkin DH Billimek J Greenfield S Kaplan SH. The effects of financial pressures on adherence and glucose control among racial/ethnically diverse patients with diabetes. J Gen Intern Med. (2012) 27:432–7. doi: 10.1007/s11606-011-1910-7, 22005941 PMC3304038

[43] Kurani SS Lampman MA Funni SA Giblon RE Inselman JW Shah ND . Area-level socioeconomic deprivation and diabetes care quality. JAMA Netw Open. (2021) 4:e2138438. doi: 10.1001/jamanetworkopen.2021.38438 34964856 PMC8717098

[44] Scott A Chambers D Goyder E O'Cathain A. Socioeconomic inequalities in adults with type 1 diabetes: a systematic review. PLoS One. (2017) 12:e0177210. doi: 10.1371/journal.pone.0177210 28489876 PMC5425027

[45] Tatulashvili S Fagherazzi G Dow C Cohen R Fosse S Bihan H. Socioeconomic inequalities and type 2 diabetes complications: a systematic review. Diabetes Metab. (2020) 46:89–99. doi: 10.1016/j.diabet.2019.11.001, 31759171

[46] Lindner LME Rathmann W Rosenbauer J. Inequalities in glycaemic control, hypoglycaemia and diabetic ketoacidosis according to socio-economic status and area-level deprivation in type 1 diabetes mellitus: a systematic review. Diabet Med. (2018) 35:12–32. doi: 10.1111/dme.13519, 28945942

[47] Greenwood J Zurek KI Grimm JM Wi C-I Vogel JT Garrison GM. Association of a housing-based individual socioeconomic status measure with diabetic control in primary care practices. Prim Care Diabetes. (2022) 16:78–83. doi: 10.1016/j.pcd.2021.10.001, 34802978

[48] Keene DE Guo M Murillo S. Housing access and diabetes self-management among low-income adults. Soc Sci Med. (2018) 197:71–7. doi: 10.1016/j.socscimed.2017.11.051, 29222997 PMC5771430

[49] Darvishi A Nikkhah A Mahmudimanesh M Balajam NZ Shafiee G Heshmat R. Socioeconomic inequalities in type 2 diabetes mellitus: a study based on a population-based survey in Iran. BMC Public Health. (2024) 24:926. doi: 10.1186/s12889-024-18452-7, 38555434 PMC10981331

[50] de Carvalho AC Silva MT Treptow ILG Latorraca COC Pacheco RL Valsecchi VADS . Health literacy in patients with type 2 diabetes mellitus: a systematic review. Clinics. (2025) 80:100774. doi: 10.1016/j.clinsp.2025.100774, 40986970 PMC12494563

[51] Correia JC Wac K Joly C Assal J-P Joshi S Fakih El Khoury C . Therapeutic patient education in the digital era: opportunities and challenges in diabetes care. Mayo Clinic Proceedings. (2025) 3:100297. doi: 10.1016/j.mcpdig.2025.100297, 41282582 PMC12634287

[52] Pennanen M Broms U Korhonen T Haukkala A Partonen T Tuulio-Henriksson A . Smoking, nicotine dependence and nicotine intake by socio-economic status and marital status. Addict Behav. (2014) 39:1145–51. doi: 10.1016/j.addbeh.2014.03.005, 24727110

[53] Zhou J Li H Xia C Shi W Wang J Chen C . How a healthy lifestyle mediates socioeconomic inequities in healthy ageing and life expectancies: a 20-year cohort of older adults. Fundam Res. (2025):100297. doi: 10.1016/j.fmre.2025.10.004

[54] Gkrinia N Belančić A. Epidemiology, health disparities, and social determinants of type 2 diabetes: a narrative review. Diabetology. (2025) 6:25. doi: 10.3390/diabetology6040025

