# Supplementary material for: A systematic review and meta-analysis of the association between low socioeconomic status and all-cause mortality in patients with diabetes
Source: Front Public Health. 2026 Jun 26;14:1841893. doi: 10.3389/fpubh.2026.1841893 (PMC13350021; doi:10.3389/fpubh.2026.1841893)

Table S1. Literature search strategy.

Table S1 Literature searching strategy

Databases	Searching strategy	Number of literature
Pubmed	((((((((((((((((((((((((((((((((((((Diabetes Mellitus[MeSH Terms]) OR (Diabetes Mellitus, Type 2[MeSH Terms])) OR (Diabetes Mellitus[Title/Abstract])) OR (Diabetes Mellitus, Type 2[Title/Abstract])) OR (Diabetes Mellitus, Stable[Title/Abstract])) OR (Stable Diabetes Mellitus[Title/Abstract])) OR (Diabetes Mellitus, Noninsulin Dependent[Title/Abstract])) OR (Diabetes Mellitus, Adult-Onset[Title/Abstract])) OR (Adult-Onset Diabetes Mellitus[Title/Abstract])) OR (Diabetes Mellitus, Adult Onset[Title/Abstract])) OR (Diabetes Mellitus, Ketosis-Resistant[Title/Abstract])) OR (Diabetes Mellitus, Ketosis Resistant[Title/Abstract])) OR (Ketosis-Resistant Diabetes Mellitus[Title/Abstract])) OR (Diabetes Mellitus, Non Insulin Dependent[Title/Abstract])) OR (Diabetes Mellitus, Non-Insulin-Dependent[Title/Abstract])) OR (Non-Insulin-Dependent Diabetes Mellitus[Title/Abstract])) OR (Diabetes Mellitus, Type II[Title/Abstract])) OR (NIDDM[Title/Abstract])) OR (Diabetes Mellitus, Maturity-Onset[Title/Abstract])) OR (Diabetes Mellitus, Maturity Onset[Title/Abstract])) OR (Maturity-Onset Diabetes Mellitus[Title/Abstract])) OR (Maturity Onset Diabetes Mellitus[Title/Abstract])) OR (MODY[Title/Abstract])) OR (Diabetes Mellitus, Slow-Onset[Title/Abstract])) OR (Diabetes Mellitus, Slow Onset[Title/Abstract])) OR (Slow-Onset Diabetes Mellitus[Title/Abstract])) OR (Type 2 Diabetes Mellitus[Title/Abstract])) OR (Noninsulin-Dependent Diabetes Mellitus[Title/Abstract])) OR (Noninsulin Dependent Diabetes Mellitus[Title/Abstract])) OR (Maturity-Onset Diabetes[Title/Abstract])) OR (Diabetes, Maturity-Onset[Title/Abstract])) OR (Maturity	1317

Onset Diabetes[Title/Abstract])) OR (Type 2 Diabetes[Title/Abstract])) OR (Diabetes, Type 2[Title/Abstract])) OR (Diabetes Mellitus, Noninsulin-Dependent[Title/Abstract])) AND ((((((((((((((((((((((Socioeconomic Factors[MeSH Terms]) OR (Socioeconomic Factors[Title/Abstract])) OR (Factor, Socioeconomic[Title/Abstract])) OR (Socioeconomic Factor[Title/Abstract])) OR (Social[Title/Abstract] AND Economic Factors[Title/Abstract])) OR (Economic[Title/Abstract] AND Social Factors[Title/Abstract])) OR (Socioeconomic Characteristics[Title/Abstract])) OR (Characteristic, Socioeconomic[Title/Abstract])) OR (Socioeconomic Characteristic[Title/Abstract])) OR (Factors, Socioeconomic[Title/Abstract])) OR (High-Income Population[Title/Abstract])) OR (High Income Population[Title/Abstract])) OR (High-Income Populations[Title/Abstract])) OR (Population, High-Income[Title/Abstract])) OR (Land Tenure[Title/Abstract])) OR (Tenure, Land[Title/Abstract])) OR (Standard of Living[Title/Abstract])) OR (Living Standard[Title/Abstract])) OR (Living Standards[Title/Abstract])) OR (Social Inequality[Title/Abstract])) OR (Inequality, Social[Title/Abstract])) OR (Social Inequalities[Title/Abstract]))) AND (((Mortality[MeSH Terms]) OR (Mortality[Title/Abstract])) OR (Mortalities[Title/Abstract])) OR (Mortality Rate[Title/Abstract])) OR (Mortality Rates[Title/Abstract])) OR (Rate, Mortality[Title/Abstract])) OR (Death Rate[Title/Abstract])) OR (Death Rates[Title/Abstract])) OR (Rate, Death[Title/Abstract])) OR (Mortality, Differential[Title/Abstract])) OR (Differential Mortality[Title/Abstract])) OR (Differential Mortalities[Title/Abstract])) OR (Mortality, Excess[Title/Abstract])) OR (Excess Mortality[Title/Abstract])) OR

	(Excess Mortalities[Title/Abstract])) OR (Mortality Determinants[Title/Abstract])) OR (Determinants, Mortality[Title/Abstract])) OR (Determinant, Mortality[Title/Abstract])) OR (Mortality Determinant[Title/Abstract])) OR (Case Fatality Rate[Title/Abstract])) OR (Case Fatality Rates[Title/Abstract])) OR (Rate, Case Fatality[Title/Abstract])) OR (Rates, Case Fatality[Title/Abstract])) OR (CFR Case Fatality Rate[Title/Abstract])) OR (Decline, Mortality[Title/Abstract])) OR (Mortality Declines[Title/Abstract])) OR (Mortality Decline[Title/Abstract])) OR (Age-Specific Death Rate[Title/Abstract])) OR (Age-Specific Death Rates[Title/Abstract])) OR (Death Rate, Age-Specific[Title/Abstract])) OR (Rate, Age-Specific Death[Title/Abstract])) OR (Age Specific Death Rate[Title/Abstract])) OR (Crude Death Rate[Title/Abstract])) OR (Crude Death Rates[Title/Abstract])) OR (Death Rate, Crude[Title/Abstract])) OR (Rate, Crude Death[Title/Abstract])) OR (Crude Mortality Rate[Title/Abstract])) OR (Crude Mortality Rates[Title/Abstract])) OR (Mortality Rate, Crude[Title/Abstract])) OR (Rate, Crude Mortality[Title/Abstract]))	
Web of science	1 "TS=(Diabetes Mellitus OR Diabetes Mellitus, Type 2 OR Diabetes Mellitus, Stable OR Stable Diabetes Mellitus OR Diabetes Mellitus, Noninsulin Dependent OR Diabetes Mellitus, Adult-Onset OR Adult-Onset Diabetes Mellitus OR Diabetes Mellitus, Adult Onset OR Diabetes Mellitus, Ketosis-Resistant OR Diabetes Mellitus, Ketosis Resistant OR Ketosis-Resistant Diabetes Mellitus OR Diabetes Mellitus, Non Insulin Dependent OR Diabetes Mellitus, Non-Insulin-Dependent OR Non-Insulin-Dependent Diabetes Mellitus OR Diabetes Mellitus, Type II NIDDM OR Diabetes Mellitus, Maturity-Onset OR Diabetes Mellitus, Maturity Onset OR Maturity-Onset Diabetes Mellitus OR Maturity Onset Diabetes Mellitus OR MODY OR	1794

Diabetes Mellitus, Slow-Onset OR Diabetes Mellitus, Slow Onset OR Slow-Onset Diabetes Mellitus OR Type 2 Diabetes Mellitus OR Noninsulin-Dependent Diabetes Mellitus OR Noninsulin Dependent Diabetes Mellitus OR Maturity-Onset Diabetes OR Diabetes, Maturity-Onset OR Maturity Onset Diabetes OR Type 2 Diabetes OR Diabetes, Type 2 OR Diabetes Mellitus, Noninsulin-Dependent) "

2 "TS=(Socioeconomic Factors OR Factor, Socioeconomic OR Socioeconomic Factor OR Social and Economic Factors OR Economic and Social Factors OR Socioeconomic Characteristics OR Characteristic, Socioeconomic OR Socioeconomic Characteristic OR Factors, Socioeconomic OR High-Income Population OR High Income Population OR High-Income Populations OR Population, High-Income OR Land Tenure OR Tenure, Land OR Standard of Living OR Living Standard OR Living Standards OR Social Inequality OR Inequality, Social OR Social Inequalities)
"

3 "TS=(Mortality OR Mortalities OR Mortality Rate OR Mortality Rates OR Rate, Mortality OR Death Rate OR Death Rates OR Rate, Death OR Mortality, Differential OR Differential Mortality OR Differential Mortalities OR Mortality, Excess OR Excess Mortality OR Excess Mortalities OR Mortality Determinants OR Determinants, Mortality OR Determinant, Mortality OR Mortality Determinant OR Case Fatality Rate OR Case Fatality Rates OR Rate, Case Fatality OR Rates, Case Fatality OR CFR Case Fatality Rate OR Decline, Mortality OR Mortality Declines OR Mortality Decline OR Age-Specific Death Rate OR Age-Specific Death Rates OR Death Rate, Age-Specific OR Rate, Age-Specific Death OR Age Specific Death Rate OR Crude Death Rate OR Crude Death Rates OR Death Rate, Crude OR Rate, Crude Death OR Crude Mortality Rate OR Crude Mortality Rates OR

	Mortality Rate, Crude OR Rate, Crude Mortality) "<br>4 "#3 AND #2 AND #1<br>"	
Embase	Embase <1974 to 2025 September 09><br><br>1 Diabetes Mellitus.ab,kf,ti. 447284<br>2 Diabetes Mellitus, Type 2.ab,kf,ti. 14030<br>3 Diabetes Mellitus, Stable.ab,kf,ti. 19<br>4 Stable Diabetes Mellitus.ab,kf,ti. 25<br>5 Diabetes Mellitus, Noninsulin Dependent.ab,kf,ti. 17<br>6 Diabetes Mellitus, Adult-Onset.ab,kf,ti. 29<br>7 Adult-Onset Diabetes Mellitus.ab,kf,ti. 214<br>8 Diabetes Mellitus, Adult Onset.ab,kf,ti. 29<br>9 Diabetes Mellitus, Ketosis-Resistant.ab,kf,ti. 1<br>10 Diabetes Mellitus, Ketosis Resistant.ab,kf,ti. 1<br>11 Ketosis-Resistant Diabetes Mellitus.ab,kf,ti. 2<br>12 Diabetes Mellitus, Non Insulin Dependent.ab,kf,ti. 248<br>13 Diabetes Mellitus, Non-Insulin-Dependent.ab,kf,ti. 248<br>14 Non-Insulin-Dependent Diabetes Mellitus.ab,kf,ti. 8461<br>15 Diabetes Mellitus, Type II.ab,kf,ti. 1813<br>16 NIDDM.ab,kf,ti.8444<br>17 Diabetes Mellitus, Maturity-Onset.ab,kf,ti. 19<br>18 Diabetes Mellitus, Maturity Onset.ab,kf,ti. 19<br>19 Maturity-Onset Diabetes Mellitus.ab,kf,ti. 160<br>20 Maturity Onset Diabetes Mellitus.ab,kf,ti. 160<br>21 MODY.ab,kf,ti. 3476<br>22 Diabetes Mellitus, Slow-Onset.ab,kf,ti. 2<br>23 Diabetes Mellitus, Slow Onset.ab,kf,ti. 2<br>24 Slow-Onset Diabetes Mellitus.ab,kf,ti. 0<br>25 Type 2 Diabetes Mellitus.ab,kf,ti. 117213	182

26 Noninsulin-Dependent Diabetes Mellitus.ab,kf,ti. 1134
27 Noninsulin Dependent Diabetes Mellitus.ab,kf,ti. 1134
28 Maturity-Onset Diabetes.ab,kf,ti. 3498
29 Diabetes, Maturity-Onset.ab,kf,ti. 65
30 Maturity Onset Diabetes.ab,kf,ti. 3498
31 Type 2 Diabetes.ab,kf,ti. 315727
32 Diabetes, Type 2.ab,kf,ti. 4126
33 Diabetes Mellitus, Noninsulin-Dependent.ab,kf,ti. 17
34 1 or 2 or 3 or 4 or 5 or 6 or 7 or 8 or 9 or 10 or 11 or 12 or 13 or 14 or 15 or 16 or 17 or 18 or 19 or 20 or 21 or 22 or 23 or 24 or 25 or 26 or 27 or 28 or 29 or 30 or 31 or 32 or 33 633260
35 Socioeconomic Factors.ab,kf,ti. 21770
36 Factor, Socioeconomic.ab,kf,ti. 18
37 Socioeconomic Factor.ab,kf,ti. 325
38 (Social and Economic Factors).ab,kf,ti. 4036
39 (Economic and Social Factors).ab,kf,ti. 2034
40 Socioeconomic Characteristics.ab,kf,ti. 4862
41 Characteristic, Socioeconomic.ab,kf,ti. 1
42 Socioeconomic Characteristic.ab,kf,ti. 30
43 Factors, Socioeconomic.ab,kf,ti. 658
44 High-Income Population.ab,kf,ti. 52
45 High Income Population.ab,kf,ti. 52
46 High-Income Populations.ab,kf,ti. 88
47 Population, High-Income.ab,kf,ti. 4
48 Land Tenure.ab,kf,ti. 305
49 Tenure, Land.ab,kf,ti. 4
50 Standard of Living.ab,kf,ti. 1887
51 Living Standard.ab,kf,ti. 657
52 Living Standards.ab,kf,ti.

2060
53 Social Inequality.ab,kf,ti. 2383
54 Inequality, Social.ab,kf,ti. 63
55 Social Inequalities.ab,kf,ti. 4630
56 35 or 36 or 37 or 38 or 39 or 40 or 41 or 42 or 43 or 44 or 45 or 46 or 47 or 48 or 49 or 50 or 51 or 52 or 53 or 54 or 55 43328
57 Mortality.ab,kf,ti. 1738244
58 Mortalities.ab,kf,ti. 20461
59 Mortality Rate.ab,kf,ti. 198544
60 Mortality Rates.ab,kf,ti. 133099
61 Rate, Mortality.ab,kf,ti. 1338
62 Death Rate.ab,kf,ti. 23713
63 Death Rates.ab,kf,ti. 17474
64 Rate, Death.ab,kf,ti. 318
65 Mortality, Differential.ab,kf,ti. 131
66 Differential Mortality.ab,kf,ti. 625
67 Differential Mortalities.ab,kf,ti. 4
68 Mortality, Excess.ab,kf,ti. 344
69 Excess Mortality.ab,kf,ti. 10282
70 Excess Mortalities.ab,kf,ti. 66
71 Mortality Determinants.ab,kf,ti. 146
72 Determinants, Mortality.ab,kf,ti. 4
73 Determinant, Mortality.ab,kf,ti. 1
74 Mortality Determinant.ab,kf,ti. 25
75 Case Fatality Rate.ab,kf,ti. 9880
76 Case Fatality Rates.ab,kf,ti. 3598
77 Rate, Case Fatality.ab,kf,ti. 60
78 Rates, Case Fatality.ab,kf,ti. 50
79 CFR Case Fatality Rate.ab,kf,ti. 9
80 Decline, Mortality.ab,kf,ti. 94
81 Mortality Declines.ab,kf,ti. 269

	82 Mortality Decline.ab,kf,ti. 528<br>83 Age-Specific Death Rate.ab,kf,ti. 56<br>84 Age-Specific Death Rates.ab,kf,ti. 346<br>85 Death Rate, Age-Specific.ab,kf,ti. 2<br>86 Rate, Age-Specific Death.ab,kf,ti. 2<br>87 Age Specific Death Rate.ab,kf,ti. 56<br>88 Crude Death Rate.ab,kf,ti. 387<br>89 Crude Death Rates.ab,kf,ti. 249<br>90 Death Rate, Crude.ab,kf,ti. 2<br>91 Rate, Crude Death.ab,kf,ti. 4<br>92 Crude Mortality Rate.ab,kf,ti. 1669<br>93 Crude Mortality Rates.ab,kf,ti. 1002<br>94 Mortality Rate, Crude.ab,kf,ti. 15<br>95 Rate, Crude Mortality.ab,kf,ti. 9<br>96 57 or 58 or 59 or 60 or 61 or 62 or 63 or 64 or 65 or 66 or 67 or 68 or 69 or 70 or 71 or 72 or 73 or 74 or 75 or 76 or 77 or 78 or 79 or 80 or 81 or 82 or 83 or 84 or 85 or 86 or 87 or 88 or 89 or 90 or 91 or 92 or 93 or 94 or 95 1777712<br>97 34 and 56 and 96 182	
Cochrane	#1 (Diabetes Mellitus):ti,ab,kw OR (Diabetes Mellitus, Type 2):ti,ab,kw OR (Diabetes Mellitus, Stable):ti,ab,kw OR (Stable Diabetes Mellitus):ti,ab,kw OR (Diabetes Mellitus, Noninsulin Dependent):ti,ab,kw 88811<br>#2 (Diabetes Mellitus, Adult-Onset):ti,ab,kw OR (Adult-Onset Diabetes Mellitus):ti,ab,kw OR (Diabetes Mellitus, Adult Onset):ti,ab,kw OR (Diabetes Mellitus, Ketosis-Resistant):ti,ab,kw OR (Diabetes Mellitus, Ketosis Resistant):ti,ab,kw 1901<br>#3 (Ketosis-Resistant Diabetes Mellitus):ti,ab,kw OR (Diabetes Mellitus, Non Insulin Dependent):ti,ab,kw OR (Diabetes	186

Mellitus, Non-Insulin-Dependent):ti,ab,kw OR (Non-Insulin-Dependent Diabetes Mellitus):ti,ab,kw OR (Diabetes Mellitus, Type II):ti,ab,kw 27978

#4 (NIDDM):ti,ab,kw OR (Diabetes Mellitus, Maturity-Onset):ti,ab,kw OR (Diabetes Mellitus, Maturity Onset):ti,ab,kw OR (Maturity-Onset Diabetes Mellitus):ti,ab,kw OR (Maturity Onset Diabetes Mellitus):ti,ab,kw 1230

#5 (MODY):ti,ab,kw OR (Diabetes Mellitus, Slow-Onset):ti,ab,kw OR (Diabetes Mellitus, Slow Onset):ti,ab,kw OR (Slow-Onset Diabetes Mellitus):ti,ab,kw OR (Type 2 Diabetes Mellitus):ti,ab,kw 57411

#6 (Noninsulin-Dependent Diabetes Mellitus):ti,ab,kw OR (Noninsulin Dependent Diabetes Mellitus):ti,ab,kw OR (Maturity-Onset Diabetes):ti,ab,kw OR (Diabetes, Maturity-Onset):ti,ab,kw OR (Maturity Onset Diabetes):ti,ab,kw 796

#7 (Type 2 Diabetes):ti,ab,kw OR (Diabetes, Type 2):ti,ab,kw OR (Diabetes Mellitus, Noninsulin-Dependent):ti,ab,kw 66101

#8 #1 OR #2 OR #3 OR #4 OR #5 OR #6 OR #7 97535

#9 (Socioeconomic Factors):ti,ab,kw OR (Factor, Socioeconomic):ti,ab,kw OR (Socioeconomic Factor):ti,ab,kw OR (Social and Economic Factors):ti,ab,kw OR (Economic and Social Factors):ti,ab,kw 8459

#10 (Socioeconomic Characteristics):ti,ab,kw OR (Characteristic, Socioeconomic):ti,ab,kw OR (Socioeconomic Characteristic):ti,ab,kw OR (Factors, Socioeconomic):ti,ab,kw OR (High-Income Population):ti,ab,kw 8306

#11 (High Income Population):ti,ab,kw OR (High-Income Populations):ti,ab,kw OR (Population, High-Income):ti,ab,kw OR (Land Tenure):ti,ab,kw OR (Tenure, Land):ti,ab,kw 1768

#12 (Standard of Living):ti,ab,kw OR (Living Standard):ti,ab,kw OR (Living Standards):ti,ab,kw OR (Social

Inequality):ti,ab,kw OR (Inequality, Social):ti,ab,kw 8110
#13 (Social Inequalities):ti,ab,kw 270
#14 #9 OR #10 OR #11 OR #12 OR #13 18623
#15 (Mortality):ti,ab,kw OR (Mortalities):ti,ab,kw OR (Mortality Rate):ti,ab,kw OR (Mortality Rates):ti,ab,kw OR (Rate, Mortality):ti,ab,kw 128533
#16 (Death Rate):ti,ab,kw OR (Death Rates):ti,ab,kw OR (Rate, Death):ti,ab,kw OR (Mortality, Differential):ti,ab,kw OR (Differential Mortality):ti,ab,kw 41223
#17 (Differential Mortalities):ti,ab,kw OR (Mortality, Excess):ti,ab,kw OR (Excess Mortality):ti,ab,kw OR (Excess Mortalities):ti,ab,kw OR (Mortality Determinants):ti,ab,kw 2716
#18 (Determinants, Mortality):ti,ab,kw OR (Determinant, Mortality):ti,ab,kw OR (Mortality Determinant):ti,ab,kw OR (Case Fatality Rate):ti,ab,kw OR (Case Fatality Rates):ti,ab,kw 1996
#19 (Rate, Case Fatality):ti,ab,kw OR (Rates, Case Fatality):ti,ab,kw OR (CFR Case Fatality Rate):ti,ab,kw OR (Decline, Mortality):ti,ab,kw OR (Mortality Declines):ti,ab,kw 3735
#20 (Mortality Decline):ti,ab,kw OR (Age-Specific Death Rate):ti,ab,kw OR (Age-Specific Death Rates):ti,ab,kw OR (Death Rate, Age-Specific):ti,ab,kw OR (Rate, Age-Specific Death):ti,ab,kw 2756
#21 (Age Specific Death Rate):ti,ab,kw OR (Crude Death Rate):ti,ab,kw OR (Crude Death Rates):ti,ab,kw OR (Death Rate, Crude):ti,ab,kw OR (Rate, Crude Death):ti,ab,kw 1476
#22 (Crude Mortality Rate):ti,ab,kw OR (Crude Mortality Rates):ti,ab,kw OR (Mortality Rate, Crude):ti,ab,kw OR (Rate, Crude Mortality):ti,ab,kw 320
#23 #15 OR #16 OR #16 OR #17 OR #18 OR #19 OR #20 OR #21 OR #22 152006
#24 #8 AND #14 AND #23 217

Figure S1A. Subgroup analysis of income-based socioeconomic status and all-cause mortality stratified by country.

Study or Subgroup	log[Odds Ratio]	SE	Weight	Odds Ratio IV, Random, 95% CI
**1.1.1 Canada**				
Rosella 2023	0.0488	0.0049	6.2%	1.05 [1.04, 1.06]
Shulman 2018	0.708	0.2989	3.8%	2.03 [1.13, 3.65]
**Subtotal (95% CI)**			**10.1%**	**1.36 [0.73, 2.57]**
Heterogeneity: Tau^2 = 0.17; Chi^2 = 4.86, df = 1 (P = 0.03); I^2 = 79%				
Test for overall effect: Z = 0.96 (P = 0.34)				
**1.1.2 China**				
Liao 2023	0.6366	0.0109	6.2%	1.89 [1.85, 1.93]
Li 2025	1.0225	0.1969	4.9%	2.78 [1.89, 4.09]
**Subtotal (95% CI)**			**11.1%**	**2.18 [1.51, 3.14]**
Heterogeneity: Tau^2 = 0.06; Chi^2 = 3.83, df = 1 (P = 0.05); I^2 = 74%				
Test for overall effect: Z = 4.18 (P < 0.0001)				
**1.1.3 Denmark**				
Dalsgaard 2015Ⅲ_M	0.3507	0.045	6.1%	1.42 [1.30, 1.55]
Dalsgaard 2015Ⅳ_F	0.3365	0.0538	6.1%	1.40 [1.26, 1.56]
**Subtotal (95% CI)**			**12.2%**	**1.41 [1.32, 1.51]**
Heterogeneity: Tau^2 = 0.00; Chi^2 = 0.04, df = 1 (P = 0.84); I^2 = 0%				
Test for overall effect: Z = 9.99 (P < 0.00001)				
**1.1.4 Durham**				
Lusk 2022	0.1989	0.0482	6.1%	1.22 [1.11, 1.34]
**Subtotal (95% CI)**			**6.1%**	**1.22 [1.11, 1.34]**
Heterogeneity: Not applicable				
Test for overall effect: Z = 4.13 (P < 0.0001)				
**1.1.5 Hyattsville**				
Saydah 2010Ⅱ	1.0784	0.0766	6.0%	2.94 [2.53, 3.42]
**Subtotal (95% CI)**			**6.0%**	**2.94 [2.53, 3.42]**
Heterogeneity: Not applicable				
Test for overall effect: Z = 14.08 (P < 0.00001)				
**1.1.6 Korea**				
Lee 2023	-0.1165	0.0544	6.1%	0.89 [0.80, 0.99]
Shin 2016Ⅰ_M	0.4447	0.1424	5.5%	1.56 [1.18, 2.06]
Shin 2016Ⅱ_F	0.4762	0.1673	5.2%	1.61 [1.16, 2.23]
Shin 2018Ⅰ_M	0.2776	0.036	6.2%	1.32 [1.23, 1.42]
Shin 2018Ⅱ_F	0.1906	0.0486	6.1%	1.21 [1.10, 1.33]
**Subtotal (95% CI)**			**29.0%**	**1.25 [1.03, 1.51]**
Heterogeneity: Tau^2 = 0.04; Chi^2 = 43.69, df = 4 (P < 0.00001); I^2 = 91%				
Test for overall effect: Z = 2.32 (P = 0.02)				
**1.1.7 Spain**				
Aguilar-Palacio 2017Ⅰ_M	0.077	0.3085	3.8%	1.08 [0.59, 1.98]
Aguilar-Palacio 2017Ⅱ_F	-0.478	0.4241	2.8%	0.62 [0.27, 1.42]
**Subtotal (95% CI)**			**6.5%**	**0.88 [0.52, 1.49]**
Heterogeneity: Tau^2 = 0.02; Chi^2 = 1.12, df = 1 (P = 0.29); I^2 = 11%				
Test for overall effect: Z = 0.47 (P = 0.64)				
**1.1.8 Sweden**				
Rawshani 2016Ⅰ	0.7514	0.0272	6.2%	2.12 [2.01, 2.24]
**Subtotal (95% CI)**			**6.2%**	**2.12 [2.01, 2.24]**
Heterogeneity: Not applicable				
Test for overall effect: Z = 27.63 (P < 0.00001)				
**1.1.9 UK**				
Campbell 2020Ⅰ_M	0.1906	0.1076	5.8%	1.21 [0.98, 1.49]
Campbell 2020Ⅱ_F	0.4383	0.1306	5.6%	1.55 [1.20, 2.00]
**Subtotal (95% CI)**			**11.3%**	**1.35 [1.06, 1.72]**
Heterogeneity: Tau^2 = 0.02; Chi^2 = 2.14, df = 1 (P = 0.14); I^2 = 53%				
Test for overall effect: Z = 2.46 (P = 0.01)				
**1.1.10 USA**				
Secrest 2011Ⅱ	1.1632	0.7073	1.4%	3.20 [0.80, 12.80]
**Subtotal (95% CI)**			**1.4%**	**3.20 [0.80, 12.80]**
Heterogeneity: Not applicable				
Test for overall effect: Z = 1.64 (P = 0.10)				
**Total (95% CI)**			**100.0%**	**1.49 [1.24, 1.79]**
Heterogeneity: Tau^2 = 0.14; Chi^2 = 3142.39, df = 18 (P < 0.00001); I^2 = 99%				
Test for overall effect: Z = 4.19 (P < 0.0001)				
Test for subgroup differences: Chi^2 = 215.61, df = 9 (P < 0.00001), I^2 = 95.8%				

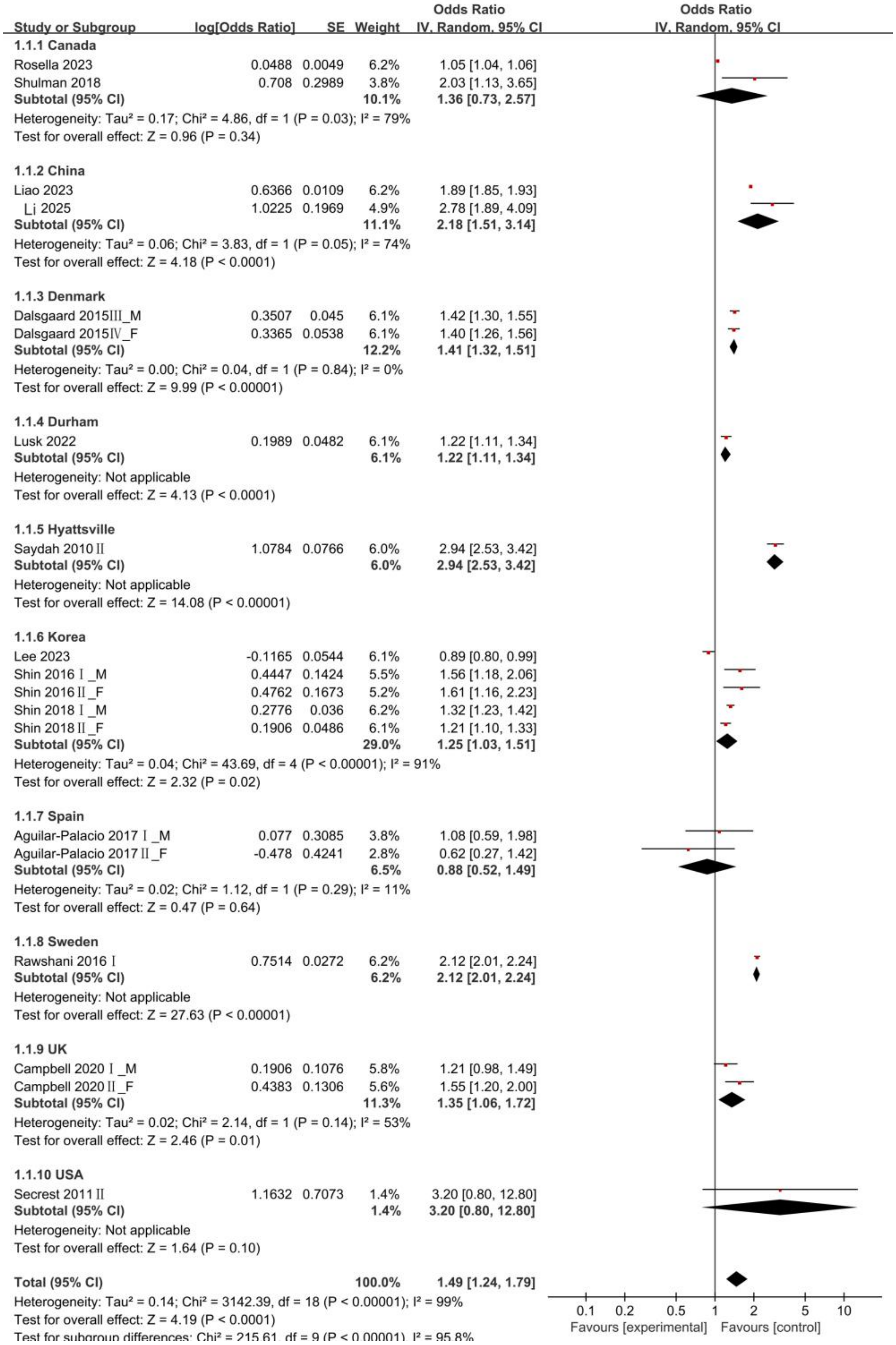

Figure S1B. Subgroup analysis of income-based socioeconomic status and all-cause mortality stratified by sex.

Study or Subgroup	log[Odds Ratio]	SE	Weight	Odds Ratio IV, Fixed, 95% CI
**1.3.1 Male**				
Aguilar-Palacio 2017Ⅰ_M	0.077	0.3085	0.5%	1.08 [0.59, 1.98]
Campbell 2020Ⅰ_M	0.1906	0.1076	3.8%	1.21 [0.98, 1.49]
Dalsgaard 2015Ⅲ_M	0.3507	0.045	21.7%	1.42 [1.30, 1.55]
Shin 2016Ⅰ_M	0.4447	0.1424	2.2%	1.56 [1.18, 2.06]
Shin 2018Ⅰ_M	0.2776	0.036	33.8%	1.32 [1.23, 1.42]
**Subtotal (95% CI)**			**61.9%**	**1.35 [1.28, 1.43]**
Heterogeneity: Chi² = 4.24, df = 4 (P = 0.37); I² = 6%				
Test for overall effect: Z = 11.35 (P < 0.00001)				
**1.3.2 Female**				
Aguilar-Palacio 2017Ⅱ_F	-0.478	0.4241	0.2%	0.62 [0.27, 1.42]
Campbell 2020Ⅱ_F	0.4383	0.1306	2.6%	1.55 [1.20, 2.00]
Dalsgaard 2015Ⅳ_F	0.3365	0.0538	15.2%	1.40 [1.26, 1.56]
Shin 2016Ⅱ_F	0.4762	0.1673	1.6%	1.61 [1.16, 2.23]
Shin 2018Ⅱ_F	0.1906	0.0486	18.6%	1.21 [1.10, 1.33]
**Subtotal (95% CI)**			**38.1%**	**1.31 [1.23, 1.40]**
Heterogeneity: Chi² = 10.48, df = 4 (P = 0.03); I² = 62%				
Test for overall effect: Z = 8.04 (P < 0.00001)				
**Total (95% CI)**			**100.0%**	**1.34 [1.28, 1.39]**
Heterogeneity: Chi² = 15.18, df = 9 (P = 0.09); I² = 41%				
Test for overall effect: Z = 13.90 (P < 0.00001)				
Test for subgroup differences: Chi² = 0.46, df = 1 (P = 0.50), I² = 0%				

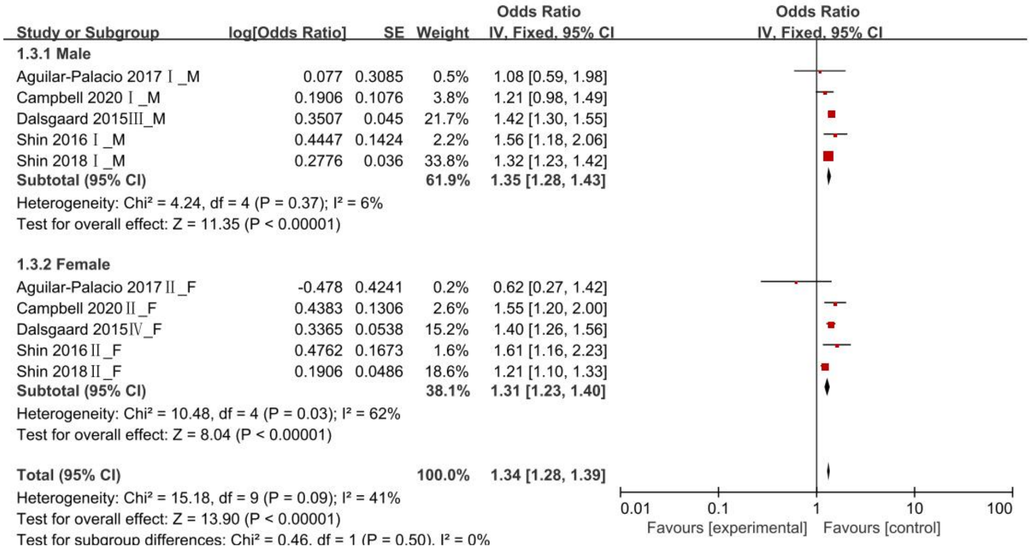

Figure S1C. Subgroup analysis of income-based socioeconomic status and all-cause mortality stratified by study design.

Study or Subgroup	log[Odds Ratio]	SE	Weight	Odds Ratio IV, Random, 95% CI
**1.2.1 Cohort**				
Aguilar-Palacio 2017Ⅰ_M	0.077	0.3085	3.8%	1.08 [0.59, 1.98]
Aguilar-Palacio 2017Ⅱ_F	-0.478	0.4241	2.8%	0.62 [0.27, 1.42]
Campbell 2020Ⅰ_M	0.1906	0.1076	5.8%	1.21 [0.98, 1.49]
Campbell 2020Ⅱ_F	0.4383	0.1306	5.6%	1.55 [1.20, 2.00]
Dalsgaard 2015Ⅲ_M	0.3507	0.045	6.1%	1.42 [1.30, 1.55]
Dalsgaard 2015Ⅳ_F	0.3365	0.0538	6.1%	1.40 [1.26, 1.56]
Lee 2023	-0.1165	0.0544	6.1%	0.89 [0.80, 0.99]
Liao 2023	0.6366	0.0109	6.2%	1.89 [1.85, 1.93]
Lusk 2022	0.1989	0.0482	6.1%	1.22 [1.11, 1.34]
Rawshani 2016Ⅰ	0.7514	0.0272	6.2%	2.12 [2.01, 2.24]
Rosella 2023	0.0488	0.0049	6.2%	1.05 [1.04, 1.06]
Secrest 2011Ⅱ	1.1632	0.7073	1.4%	3.20 [0.80, 12.80]
Shin 2016Ⅰ_M	0.4447	0.1424	5.5%	1.56 [1.18, 2.06]
Shin 2016Ⅱ_F	0.4762	0.1673	5.2%	1.61 [1.16, 2.23]
Shin 2018Ⅰ_M	0.2776	0.036	6.2%	1.32 [1.23, 1.42]
Shin 2018Ⅱ_F	0.1906	0.0486	6.1%	1.21 [1.10, 1.33]
Shulman 2018	0.708	0.2989	3.8%	2.03 [1.13, 3.65]
Li 2025	1.0225	0.1969	4.9%	2.78 [1.89, 4.09]
**Subtotal (95% CI)**			**94.0%**	**1.42 [1.18, 1.72]**
Heterogeneity: Tau² = 0.14; Chi² = 3001.55, df = 17 (P < 0.00001); I² = 99%				
Test for overall effect: Z = 3.67 (P = 0.0002)				
**1.2.2 Cross-sectional**				
Saydah 2010Ⅱ	1.0784	0.0766	6.0%	2.94 [2.53, 3.42]
**Subtotal (95% CI)**			**6.0%**	**2.94 [2.53, 3.42]**
Heterogeneity: Not applicable				
Test for overall effect: Z = 14.08 (P < 0.00001)				
**Total (95% CI)**			**100.0%**	**1.49 [1.24, 1.79]**
Heterogeneity: Tau² = 0.14; Chi² = 3142.39, df = 18 (P < 0.00001); I² = 99%				
Test for overall effect: Z = 4.19 (P < 0.0001)				
Test for subgroup differences: Chi² = 34.64, df = 1 (P < 0.00001), I² = 97.1%				

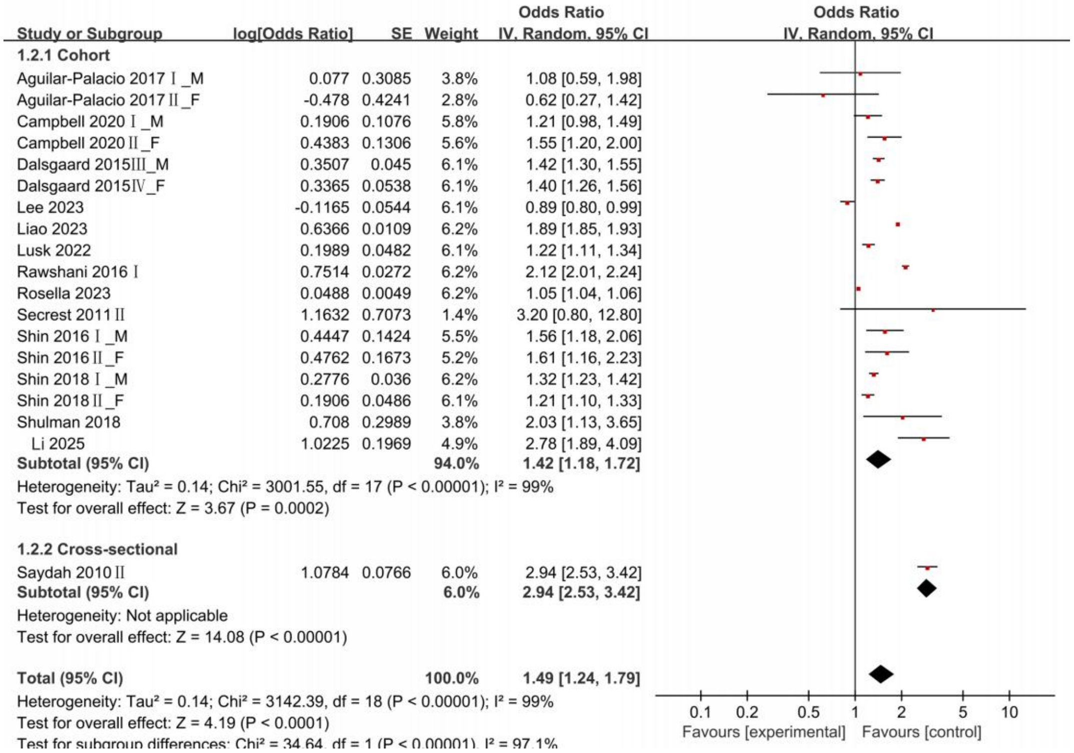

Figure S2A. Funnel plot assessing publication bias stratified by country.

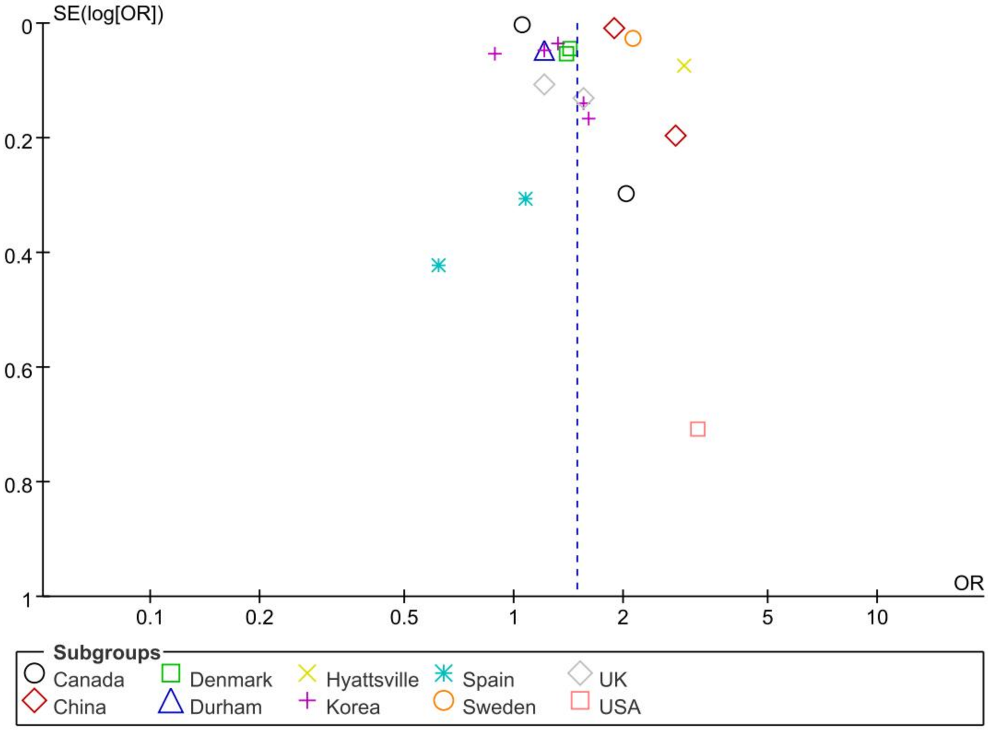

Figure S2B. Funnel plot assessing publication bias stratified by sex.

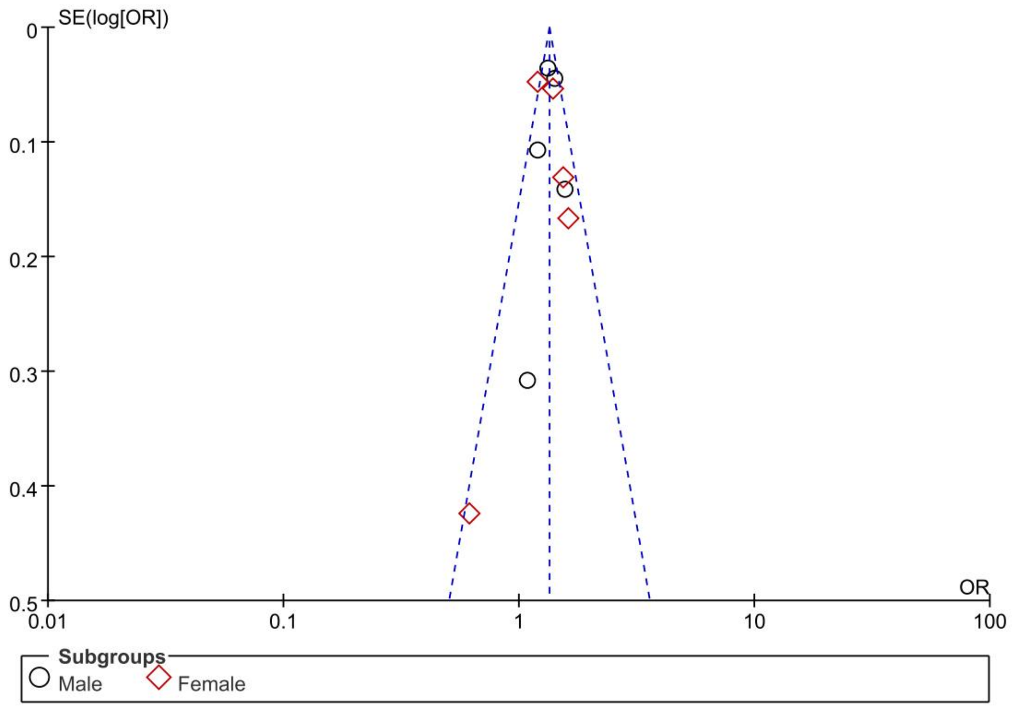

Figure S2C. Funnel plot assessing publication bias stratified by study design.

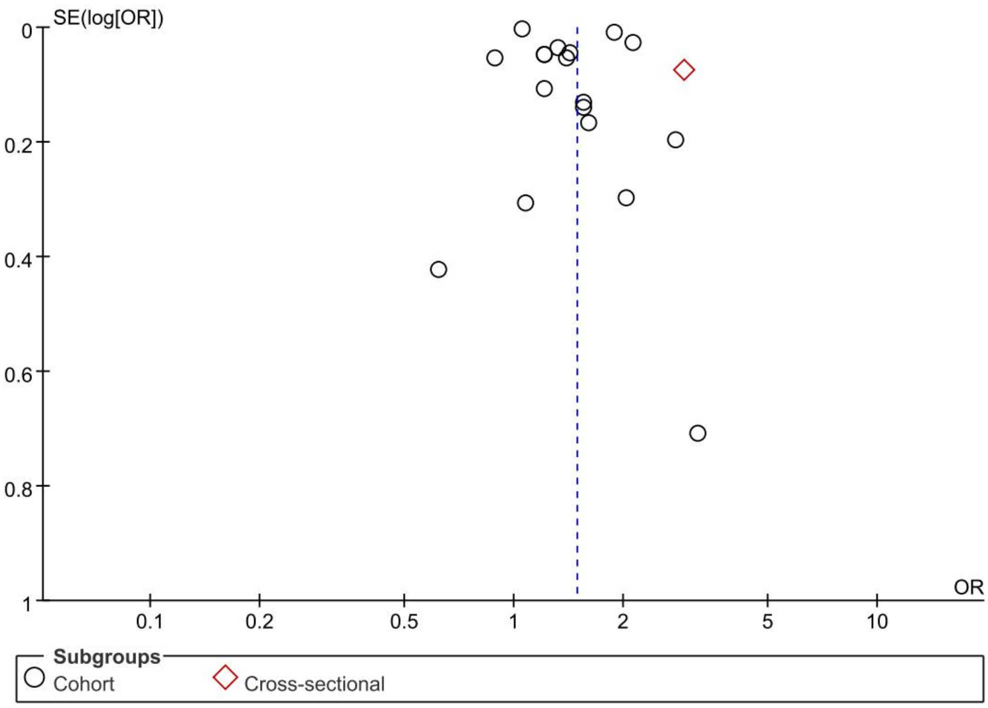

Figure S3A. Forest plot of country-specific pooled estimates for the association between low socioeconomic status and all-cause mortality.

Study or Subgroup	log[Odds Ratio]	SE	Weight	Odds Ratio IV, Random, 95% CI
**3.1.1 Australia**				
Blomster 2017	0.2927	0.0649	9.3%	1.34 [1.18, 1.52]
**Subtotal (95% CI)**			**9.3%**	**1.34 [1.18, 1.52]**
Heterogeneity: Not applicable				
Test for overall effect: Z = 4.51 (P < 0.00001)				
**3.1.2 Belgium**				
Vandenheede 2011 Ⅰ_M	0.9361	0.2129	6.2%	2.55 [1.68, 3.87]
Vandenheede 2011 Ⅱ_F	2.0162	0.3852	3.4%	7.51 [3.53, 15.98]
**Subtotal (95% CI)**			**9.5%**	**4.17 [1.45, 11.97]**
Heterogeneity: Tau² = 0.49; Chi² = 6.02, df = 1 (P = 0.01); I² = 83%				
Test for overall effect: Z = 2.66 (P = 0.008)				
**3.1.3 China**				
Liao 2023	0.6366	0.0109	9.8%	1.89 [1.85, 1.93]
**Subtotal (95% CI)**			**9.8%**	**1.89 [1.85, 1.93]**
Heterogeneity: Not applicable				
Test for overall effect: Z = 58.40 (P < 0.00001)				
**3.1.4 Denmark**				
Dalsgaard 2015 Ⅰ_M	0.1906	0.0394	9.6%	1.21 [1.12, 1.31]
Dalsgaard 2015 Ⅱ_F	0.2546	0.0498	9.5%	1.29 [1.17, 1.42]
**Subtotal (95% CI)**			**19.0%**	**1.24 [1.17, 1.32]**
Heterogeneity: Tau² = 0.00; Chi² = 1.02, df = 1 (P = 0.31); I² = 2%				
Test for overall effect: Z = 6.91 (P < 0.00001)				
**3.1.5 Hyattsville**				
Saydah 2010 Ⅰ	0.9002	0.0687	9.2%	2.46 [2.15, 2.81]
**Subtotal (95% CI)**			**9.2%**	**2.46 [2.15, 2.81]**
Heterogeneity: Not applicable				
Test for overall effect: Z = 13.10 (P < 0.00001)				
**3.1.6 Sweden**				
Rawshani 2016 Ⅱ	0.207	0.0212	9.7%	1.23 [1.18, 1.28]
**Subtotal (95% CI)**			**9.7%**	**1.23 [1.18, 1.28]**
Heterogeneity: Not applicable				
Test for overall effect: Z = 9.76 (P < 0.00001)				
**3.1.7 UK**				
Vandenheede 2013 Ⅰ_M	0.3221	0.0713	9.2%	1.38 [1.20, 1.59]
Vandenheede 2013 Ⅱ_F	0.7793	0.2962	4.6%	2.18 [1.22, 3.90]
Vandenheede 2013Ⅲ_M	0.1989	0.0814	9.0%	1.22 [1.04, 1.43]
Vandenheede 2013Ⅳ_F	0.6678	0.1238	8.2%	1.95 [1.53, 2.49]
**Subtotal (95% CI)**			**30.9%**	**1.52 [1.22, 1.90]**
Heterogeneity: Tau² = 0.03; Chi² = 12.27, df = 3 (P = 0.007); I² = 76%				
Test for overall effect: Z = 3.71 (P = 0.0002)				
**3.1.8 USA**				
Secrest 2011 Ⅰ	1.0986	0.4675	2.6%	3.00 [1.20, 7.50]
**Subtotal (95% CI)**			**2.6%**	**3.00 [1.20, 7.50]**
Heterogeneity: Not applicable				
Test for overall effect: Z = 2.35 (P = 0.02)				
**Total (95% CI)**			**100.0%**	**1.69 [1.43, 2.01]**
Heterogeneity: Tau² = 0.08; Chi² = 519.21, df = 12 (P < 0.00001); I² = 98%				
Test for overall effect: Z = 6.03 (P < 0.00001)				
Test for subgroup differences: Chi² = 475.99, df = 7 (P < 0.00001), I² = 98.5%				

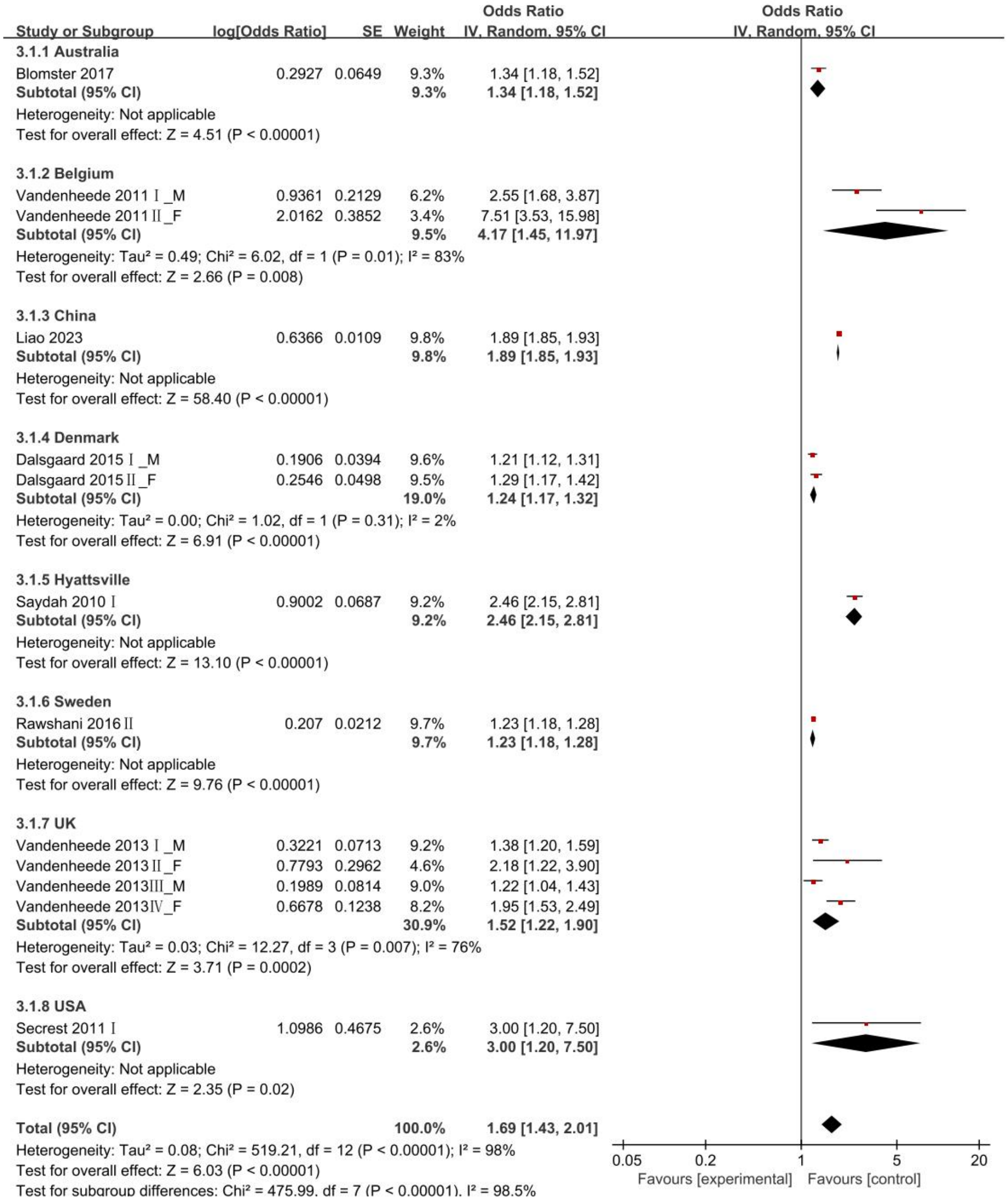

Figure S3B. Forest plot of sex-specific pooled estimates for the association between low socioeconomic status and all-cause mortality.

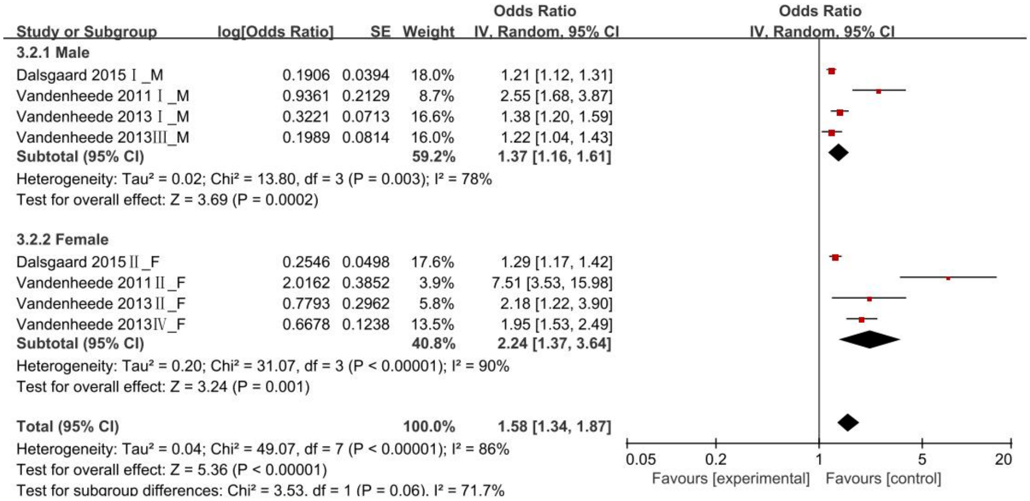

Figure S3C. Forest plot of pooled estimates stratified by study design.

Study or Subgroup	log[Odds Ratio]	SE	Weight	Odds Ratio IV, Random, 95% CI
**3.3.1 cross-sectional**				
Saydah 2010 Ⅰ	0.9002	0.0687	9.2%	2.46 [2.15, 2.81]
**Subtotal (95% CI)**			**9.2%**	**2.46 [2.15, 2.81]**
Heterogeneity: Not applicable				
Test for overall effect: Z = 13.10 (P < 0.00001)				
**3.3.2 cohort**				
Blomster 2017	0.2927	0.0649	9.3%	1.34 [1.18, 1.52]
Dalsgaard 2015 Ⅰ_M	0.1906	0.0394	9.6%	1.21 [1.12, 1.31]
Dalsgaard 2015 Ⅱ_F	0.2546	0.0498	9.5%	1.29 [1.17, 1.42]
Liao 2023	0.6366	0.0109	9.8%	1.89 [1.85, 1.93]
Rawshani 2016 Ⅱ	0.207	0.0212	9.7%	1.23 [1.18, 1.28]
Secrest 2011 Ⅰ	1.0986	0.4675	2.6%	3.00 [1.20, 7.50]
Vandenheede 2011 Ⅰ_M	0.9361	0.2129	6.2%	2.55 [1.68, 3.87]
Vandenheede 2011 Ⅱ_F	2.0162	0.3852	3.4%	7.51 [3.53, 15.98]
Vandenheede 2013 Ⅰ_M	0.3221	0.0713	9.2%	1.38 [1.20, 1.59]
Vandenheede 2013 Ⅱ_F	0.7793	0.2962	4.6%	2.18 [1.22, 3.90]
Vandenheede 2013Ⅲ_M	0.1989	0.0814	9.0%	1.22 [1.04, 1.43]
Vandenheede 2013Ⅳ_F	0.6678	0.1238	8.2%	1.95 [1.53, 2.49]
**Subtotal (95% CI)**			**90.8%**	**1.63 [1.36, 1.95]**
Heterogeneity: Tau² = 0.08; Chi² = 487.26, df = 11 (P < 0.00001); I² = 98%				
Test for overall effect: Z = 5.36 (P < 0.00001)				
**Total (95% CI)**			**100.0%**	**1.69 [1.43, 2.01]**
Heterogeneity: Tau² = 0.08; Chi² = 519.21, df = 12 (P < 0.00001); I² = 98%				
Test for overall effect: Z = 6.03 (P < 0.00001)				
Test for subgroup differences: Chi² = 13.14, df = 1 (P = 0.0003), I² = 92.4%				

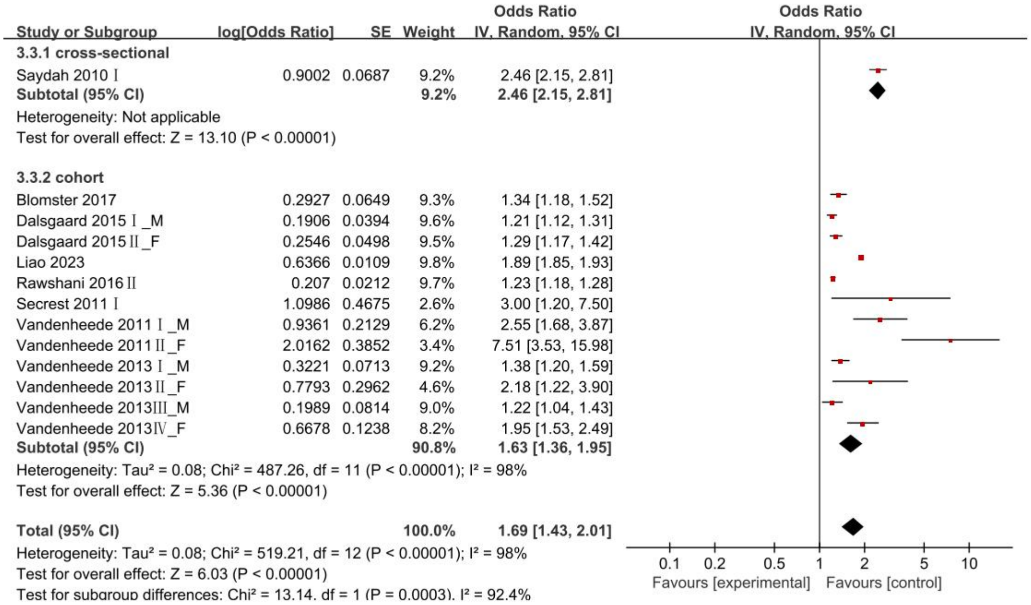

Figure S4A. Funnel plot of subgroup analysis stratified by country.

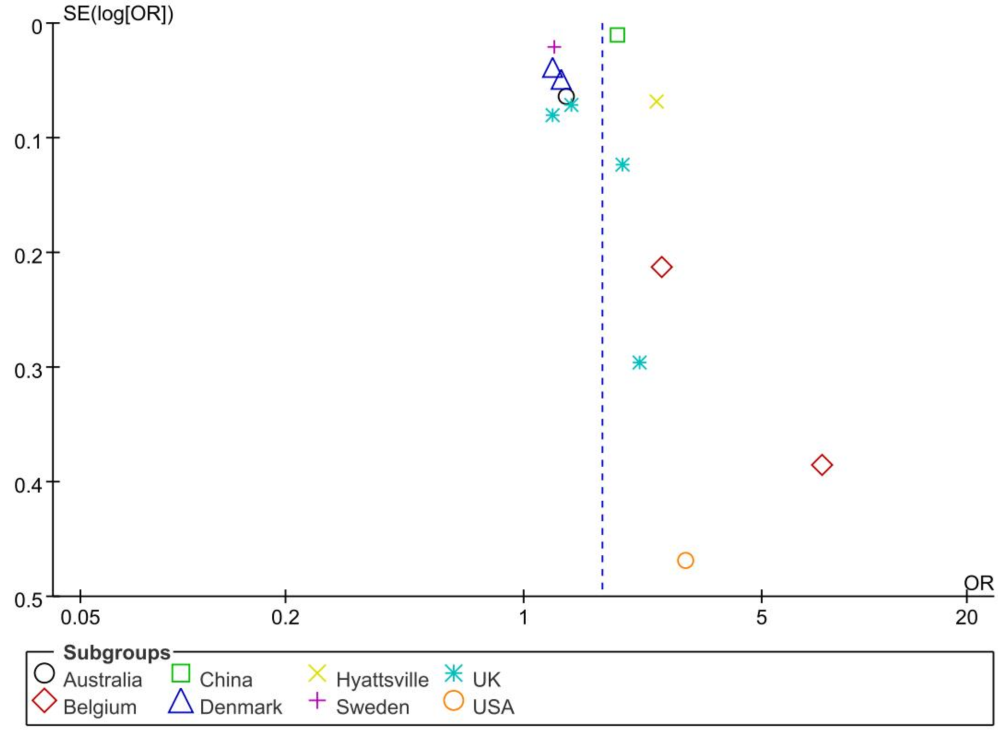

Figure S4B. Funnel plot of subgroup analysis stratified by sex.

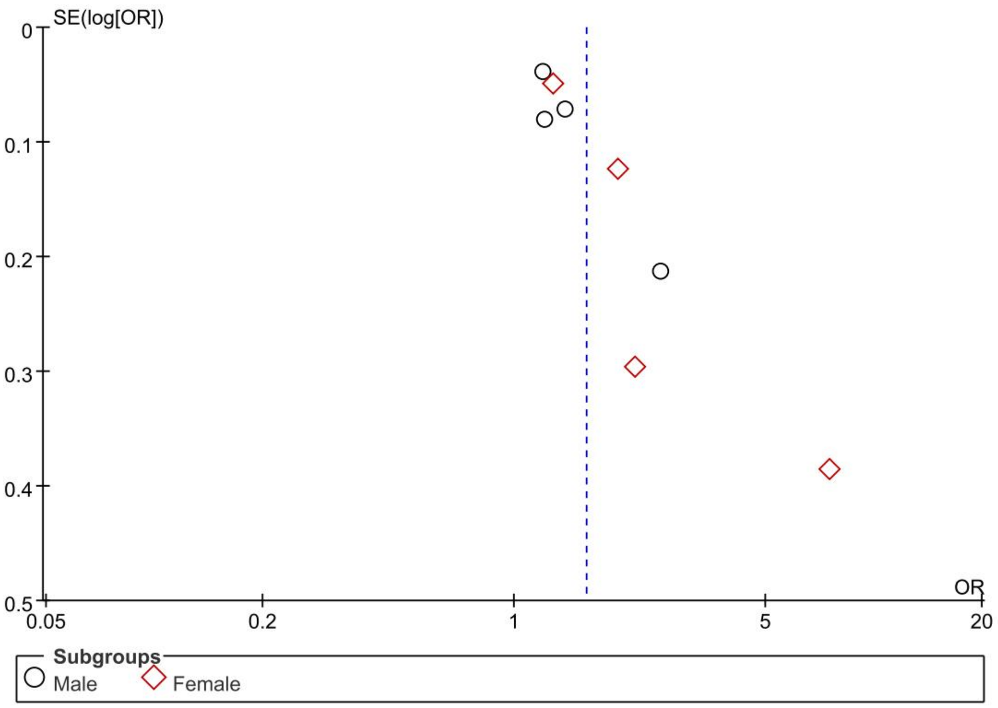

Figure S4C. Funnel plot of subgroup analysis stratified by study design.

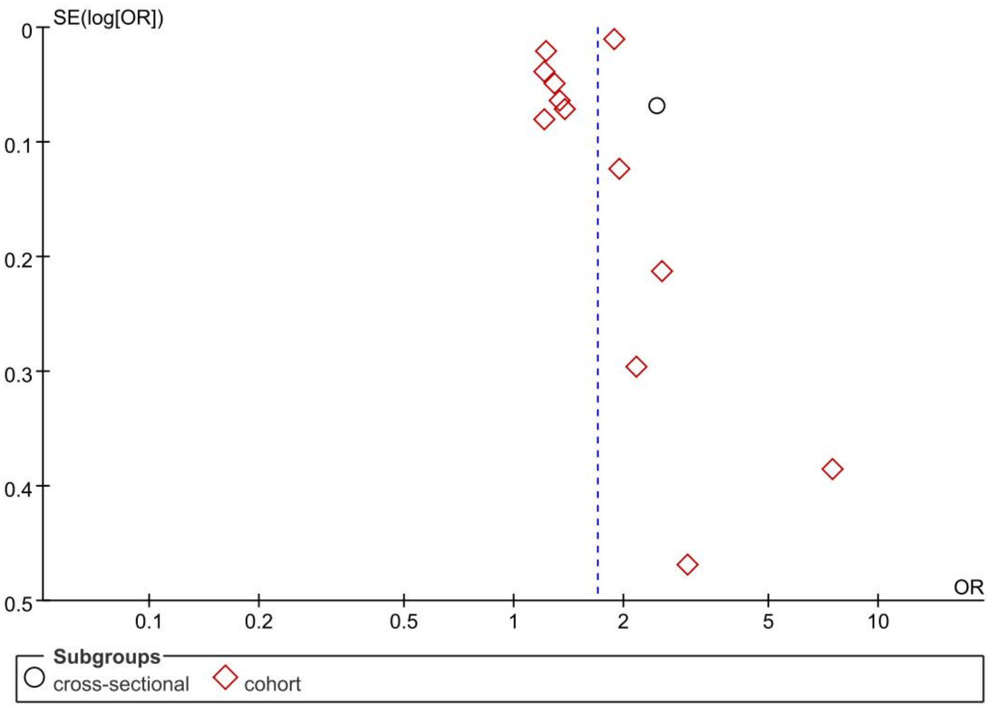

Figure S5A. Sensitivity analysis using alternative meta-analytic models, stratified by country.

Study or Subgroup	log[Odds Ratio]	SE	Weight	Odds Ratio IV, Fixed, 95% CI
**4.1.1 Belgium**				
Vandenheede 2011Ⅲ_M	0.5188	0.1934	9.4%	1.68 [1.15, 2.45]
Vandenheede 2011Ⅳ_F	1.6253	0.286	4.3%	5.08 [2.90, 8.90]
**Subtotal (95% CI)**			**13.6%**	**2.38 [1.74, 3.25]**
Heterogeneity: Chi² = 10.27, df = 1 (P = 0.001); I² = 90%				
Test for overall effect: Z = 5.41 (P < 0.00001)				
**4.1.2 UK**				
Vandenheede 2013Ⅴ_M	0.9969	0.244	5.9%	2.71 [1.68, 4.37]
Vandenheede 2013Ⅵ_F	0.94	0.0843	49.3%	2.56 [2.17, 3.02]
**Subtotal (95% CI)**			**55.1%**	**2.58 [2.20, 3.01]**
Heterogeneity: Chi² = 0.05, df = 1 (P = 0.83); I² = 0%				
Test for overall effect: Z = 11.87 (P < 0.00001)				
**4.1.3 Korea**				
Shin 2016Ⅴ_M	0.1044	0.1302	20.7%	1.11 [0.86, 1.43]
Shin 2016Ⅵ_F	0.2624	0.182	10.6%	1.30 [0.91, 1.86]
**Subtotal (95% CI)**			**31.2%**	**1.17 [0.95, 1.44]**
Heterogeneity: Chi² = 0.50, df = 1 (P = 0.48); I² = 0%				
Test for overall effect: Z = 1.49 (P = 0.14)				
**Total (95% CI)**			**100.0%**	**1.99 [1.77, 2.24]**
Heterogeneity: Chi² = 47.60, df = 5 (P < 0.00001); I² = 89%				
Test for overall effect: Z = 11.65 (P < 0.00001)				
Test for subgroup differences: Chi² = 36.79, df = 2 (P < 0.00001), I² = 94.6%				

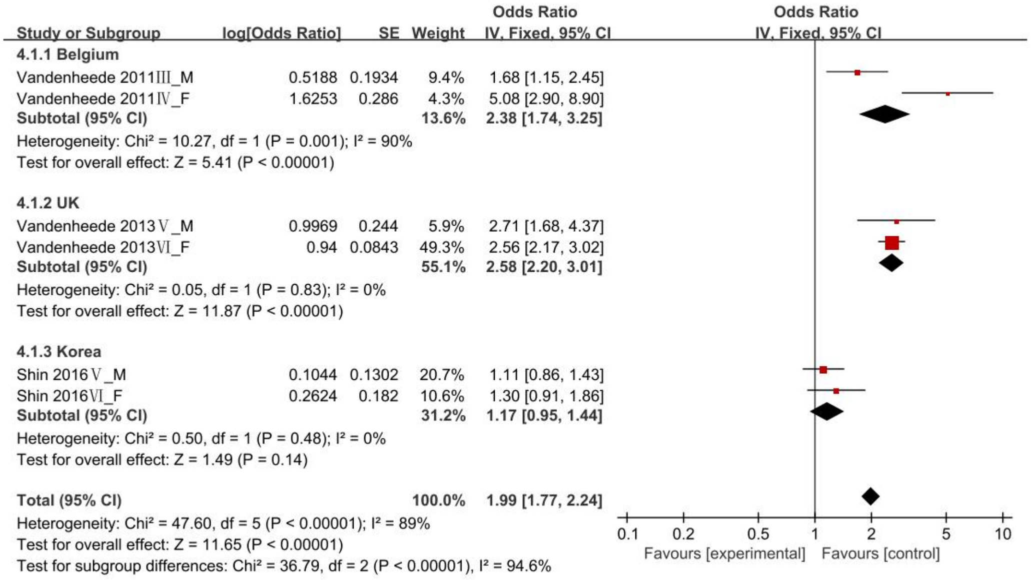

Figure S5B. Sensitivity analysis using alternative meta-analytic models, stratified by sex.

Study or Subgroup	log[Odds Ratio]	SE	Weight	Odds Ratio IV, Random, 95% CI
**4.2.1 Male**				
Shin 2016Ⅴ_M	0.1044	0.1302	18.1%	1.11 [0.86, 1.43]
Vandenheede 2011Ⅲ_M	0.5188	0.1934	16.6%	1.68 [1.15, 2.45]
Vandenheede 2013Ⅴ_M	0.9969	0.244	15.3%	2.71 [1.68, 4.37]
**Subtotal (95% CI)**			**50.0%**	**1.66 [1.01, 2.74]**
Heterogeneity: Tau² = 0.16; Chi² = 11.34, df = 2 (P = 0.003); I² = 82%				
Test for overall effect: Z = 1.99 (P = 0.05)				
**4.2.2 Female**				
Shin 2016Ⅵ_F	0.2624	0.182	16.9%	1.30 [0.91, 1.86]
Vandenheede 2011Ⅳ_F	1.6253	0.286	14.2%	5.08 [2.90, 8.90]
Vandenheede 2013Ⅵ_F	0.94	0.0843	18.9%	2.56 [2.17, 3.02]
**Subtotal (95% CI)**			**50.0%**	**2.48 [1.36, 4.51]**
Heterogeneity: Tau² = 0.24; Chi² = 18.81, df = 2 (P < 0.0001); I² = 89%				
Test for overall effect: Z = 2.97 (P = 0.003)				
**Total (95% CI)**			**100.0%**	**2.03 [1.36, 3.04]**
Heterogeneity: Tau² = 0.22; Chi² = 47.60, df = 5 (P < 0.00001); I² = 89%				
Test for overall effect: Z = 3.46 (P = 0.0006)				
Test for subgroup differences: Chi² = 1.01, df = 1 (P = 0.31), I² = 1.0%				

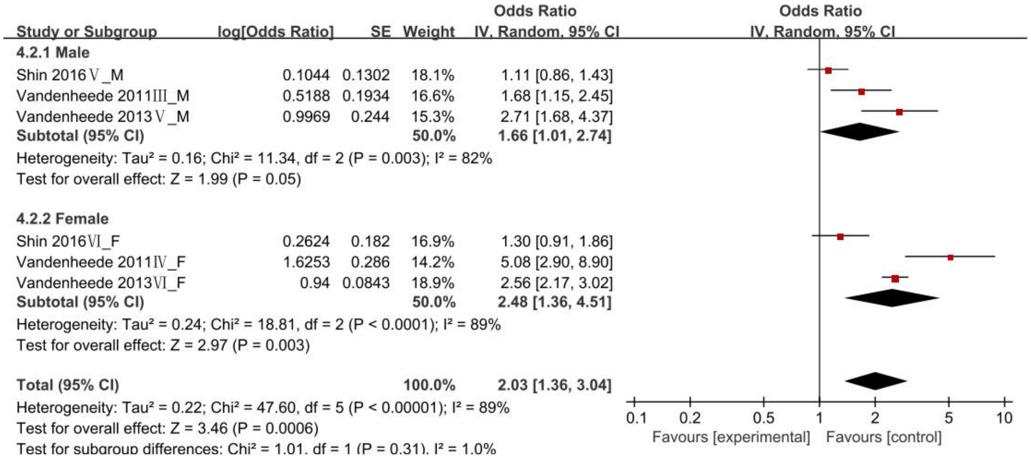

Figure S6A. Funnel plot assessing publication bias for secondary outcomes stratified by country.

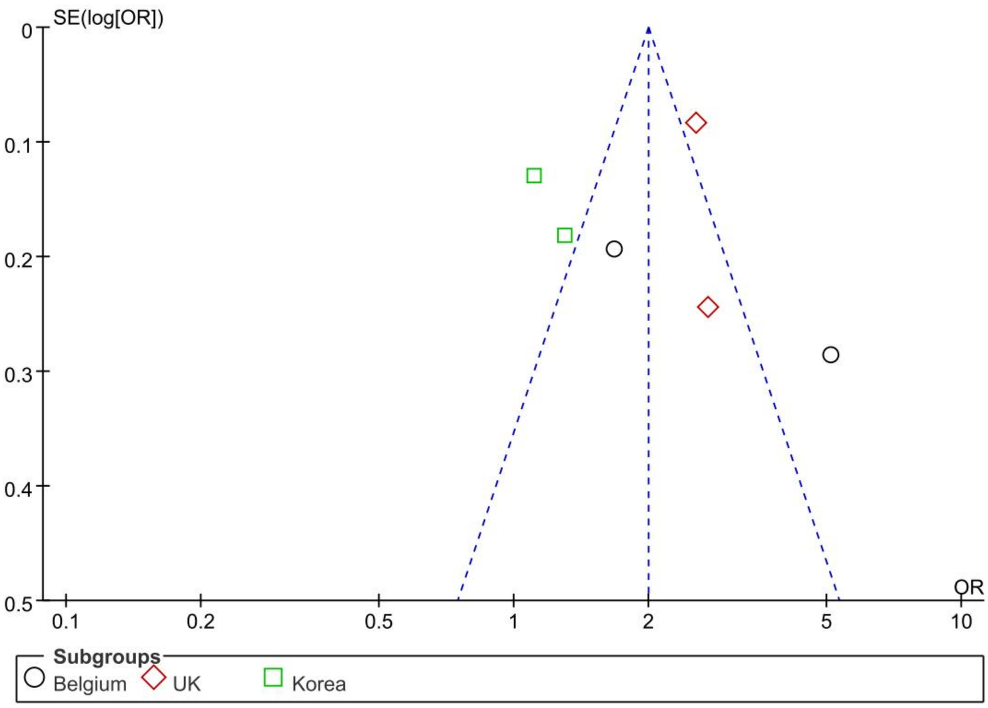

Figure S6B. Funnel plot assessing publication bias for secondary outcomes stratified by sex.

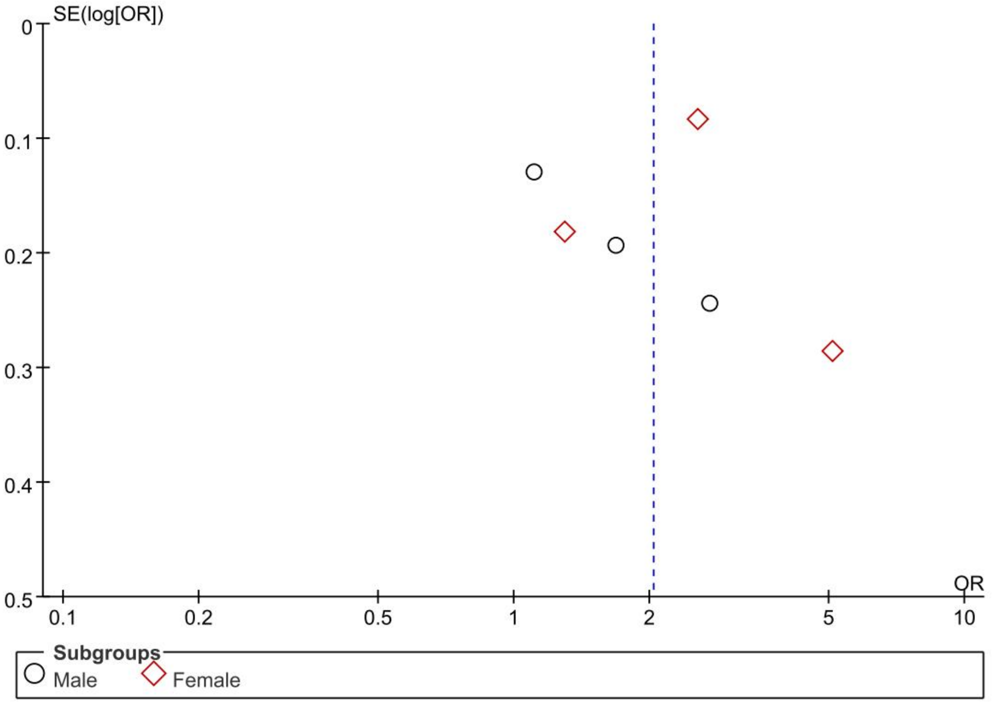

Figure S7. Forest plot of the association between socioeconomic status and cause-specific mortality in selected subgroups.

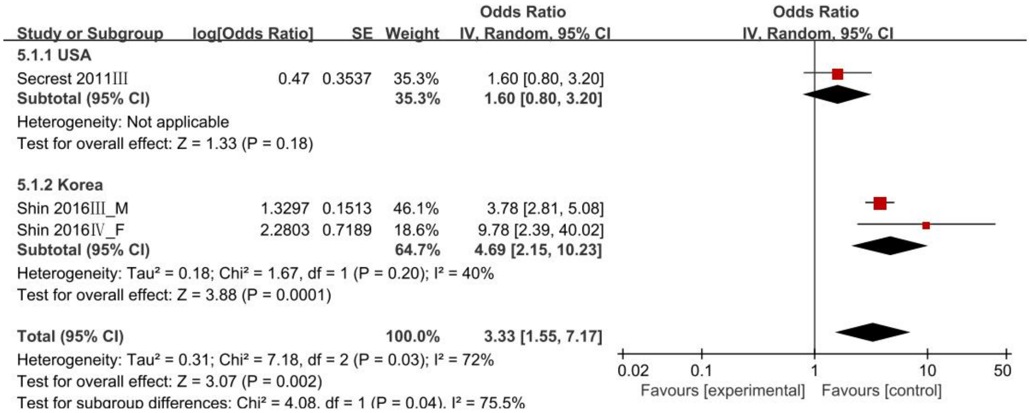

Figure S8. Funnel plot assessing publication bias in Asian and North American studies.

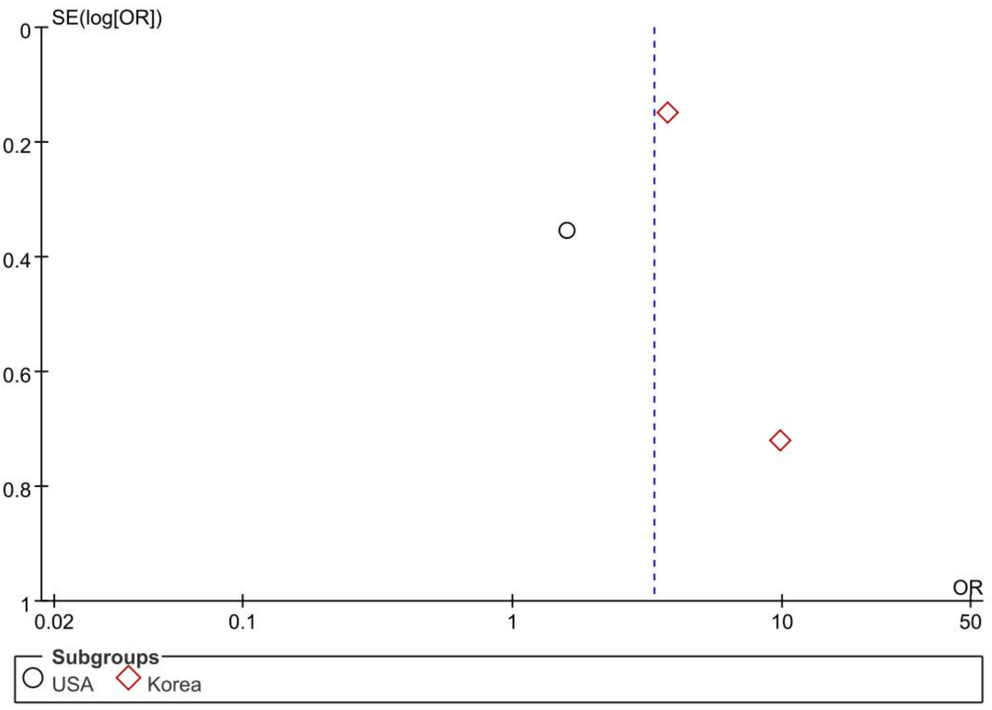

Figure S9. Leave-one-out sensitivity analysis using a random-effects model (linear scale).

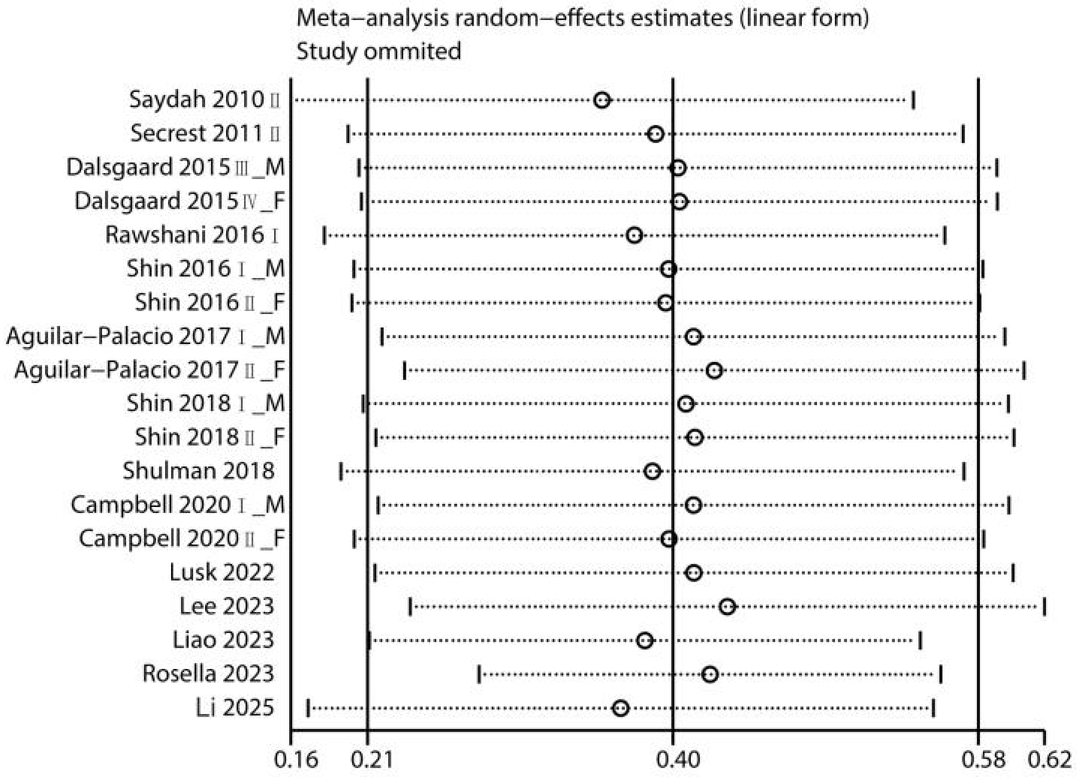

Figure S10. Leave-one-out sensitivity analysis using a fixed-effects model (linear scale).

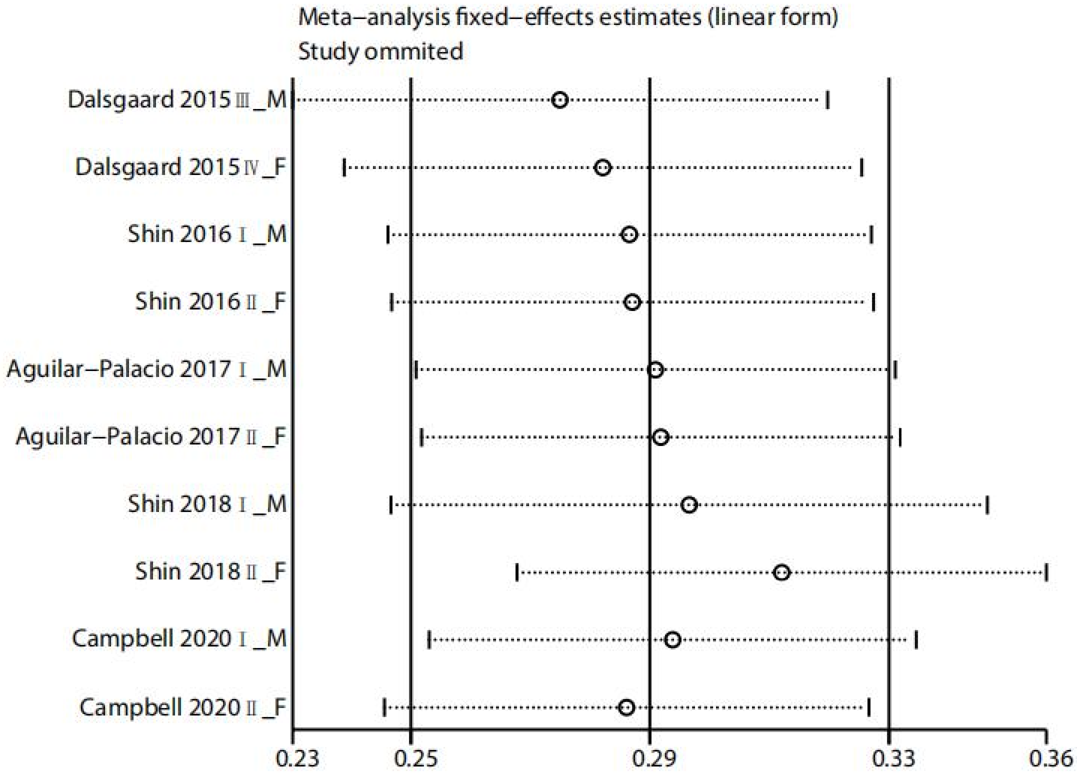

Figure S11. Leave-one-out sensitivity analysis for subgroup-specific estimates.

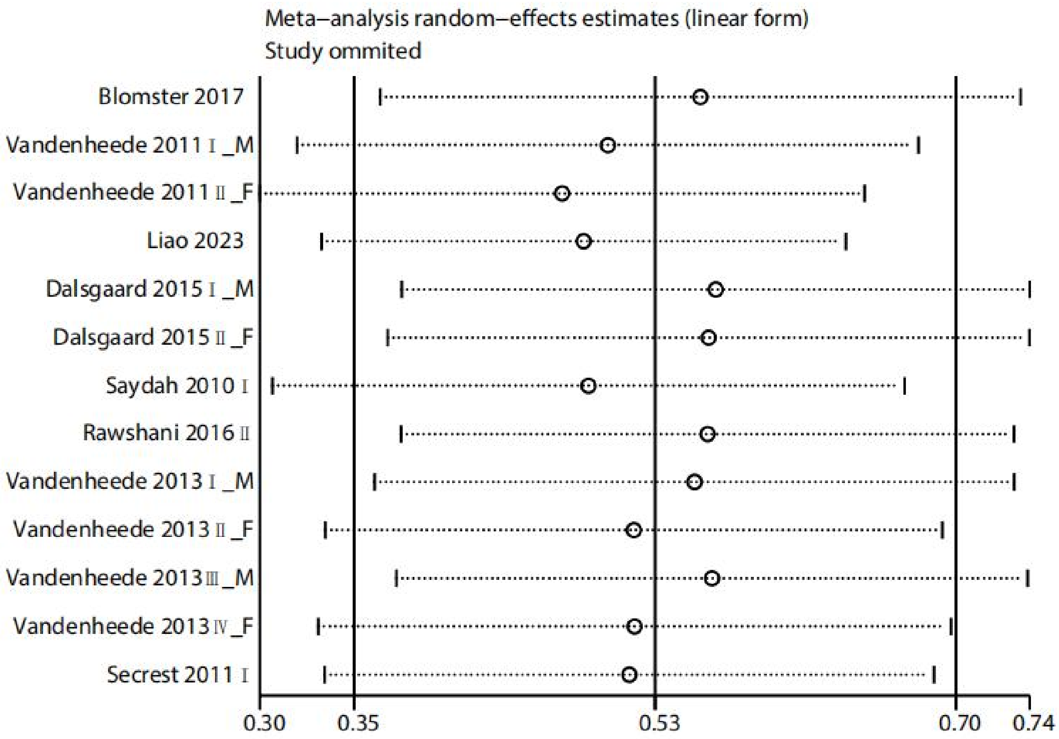

Supplement: Supplementary file 1 [file Data_Sheet_1.pdf]
